# Base excision repair causes age-dependent accumulation of single-stranded DNA breaks that contribute to Parkinson disease pathology

**DOI:** 10.1016/j.celrep.2021.109668

**Published:** 2021-09-07

**Authors:** Tanima SenGupta, Konstantinos Palikaras, Ying Q. Esbensen, Georgios Konstantinidis, Francisco Jose Naranjo Galindo, Kavya Achanta, Henok Kassahun, Ioanna Stavgiannoudaki, Vilhelm A. Bohr, Mansour Akbari, Johannes Gaare, Charalampos Tzoulis, Nektarios Tavernarakis, Hilde Nilsen

**Affiliations:** 1 Department of Clinical Molecular Biology, University of Oslo, Oslo, Norway; 2 Department of Clinical Molecular Biology, Akershus University Hospital, Lørenskog, Norway; 3 Institute of Molecular Biology and Biotechnology, Foundation for Research and Technology-Hellas, Hellas, Greece; 4 Department of Basic Sciences, Faculty of Medicine, University of Crete, Heraklion, 70013 Crete, Greece; 5 Department of Physiology, School of Medicine, National and Kapodistrian University of Athens, Athens, Greece; 6 Center for Healthy Aging, Department of Cellular and Molecular Medicine, SUND, University of Copenhagen, 2200 Copenhagen, Denmark; 7 DNA Repair Section, National Institute on Aging, 251 Bayview Boulevard, Baltimore, MD, USA; 8 Neuro-SysMed, Department of Neurology, Haukeland University Hospital, 5021 Bergen, Norway; 9 Department of Clinical Medicine, University of Bergen, Pb 7804, 5020 Bergen, Norway

**Keywords:** base excision repair, *C. elegans*, DNA-glycosylase, hydrogen peroxide, mitohormesis, NTH-1, oxidative DNA damage, Parkinson disease, aging, neurodegeneration

## Abstract

Aging, genomic stress, and mitochondrial dysfunction are risk factors for neurodegenerative pathologies, such as Parkinson disease (PD). Although genomic instability is associated with aging and mitochondrial impairment, the underlying mechanisms are poorly understood. Here, we show that base excision repair generates genomic stress, promoting age-related neurodegeneration in a *Caenorhabditis elegans* PD model. A physiological level of NTH-1 DNA glycosylase mediates mitochondrial and nuclear genomic instability, which promote degeneration of dopaminergic neurons in older nematodes. Conversely, NTH-1 deficiency protects against α-synuclein-induced neurotoxicity, maintaining neuronal function with age. This apparent paradox is caused by modulation of mitochondrial transcription in NTH-1-deficient cells, and this modulation activates LMD-3, JNK-1, and SKN-1 and induces mitohormesis. The dependance of neuroprotection on mitochondrial transcription highlights the integration of BER and transcription regulation during physiological aging. Finally, whole-exome sequencing of genomic DNA from patients with idiopathic PD suggests that base excision repair might modulate susceptibility to PD in humans.

## Highlights

• Incomplete base excision repair is a source of genomic stress during aging
• The NTH-1 DNA glycosylase is a key mediator of age-dependent genomic instability
• Compromised NTH-1 activity promotes neuroprotection in PD nematodes
• NTH-1 deficiency triggers LMD-3/JNK-1/SKN-1-dependent mitohormetic response

## Graphical abstract

**Figure:**
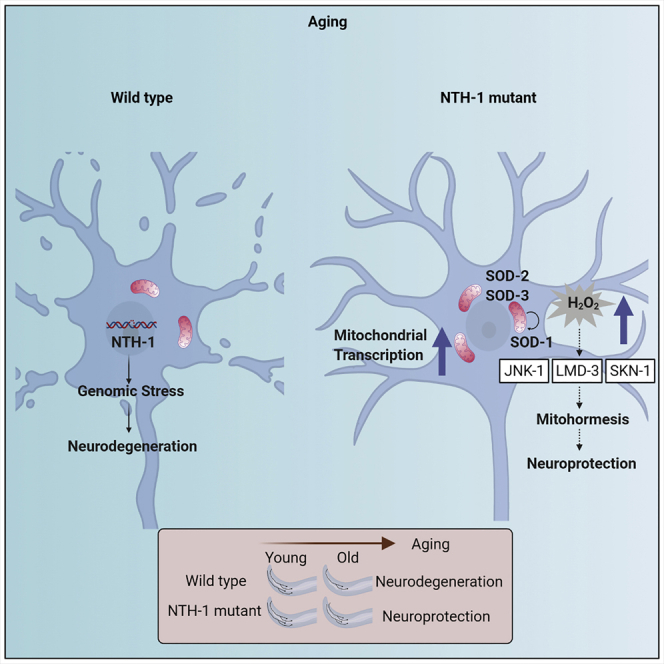

## Teaser

SenGupta et al. find that age-dependent deregulation in the base excision repair (BER) pathway affects the survival of dopaminergic neurons in a Parkinson disease (PD) nematode model. The enrichment of genetic variation in BER enzymes in patients with PD indicates that BER might serve as a pathophysiology modulator in humans.

## Introduction

Parkinson disease (PD) is the second most common neurodegenerative disorder in humans. PD primarily affects the nigrostriatal dopaminergic circuits of the brain. Clinical diagnosis of PD is based on observation of motor function defects along with significant loss of dopaminergic (DA) neurons in the substantia nigra pars compacta. Although neurodegeneration in PD is not restricted to these neurons, they are especially sensitive. All neurons are vulnerable to aging and oxidative stress, because of their high energy demand, intensive metabolism, and production of high levels of endogenous reactive oxygen species (ROS) (Camandola and Mattson, 2017). Impaired mitochondrial function is also a prominent characteristic of PD (Fukae et al., 2007; Lou et al., 2020). Thus, PD neurons experience oxidative stress that can damage cellular macromolecules, including DNA (Barzilai et al., 2017). Consistent with this, the genomic stress marker γH2AX is seen in neuronal cells from human PD patients (Sepe et al., 2016). However, the cause of aging-associated genomic stress in neurons remains elusive.

**Figure 1:**
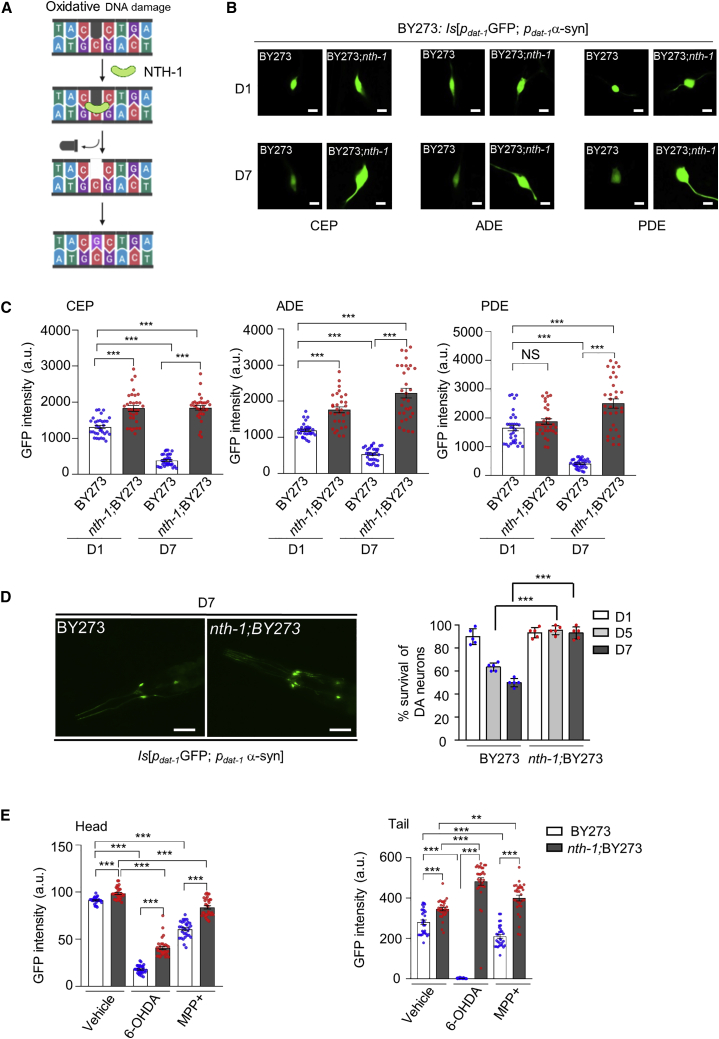
NTH-1 deficiency protects against α-synuclein neurotoxicity in a *C. elegans* Parkinson disease model (A) Schematic of the BER pathway. (B–D) Transgenic nematodes co-expressing human α-synuclein (α-syn) and cytoplasmic GFP in dopaminergic (DA) neurons display progressive degeneration with age. NTH-1 deficiency confers resistance to neuronal loss. (B) Images of the head (CEPs and ADEs) and middle body (PDEs) region of BY273 animals show age-related deterioration of DA neuronal cells. Age-dependent neurodegeneration is abolished in *nth-1;*BY273 mutants (scale bar, 5 μm, 63× objective). (C) The column scatterplot represents GFP intensity of the CEPs, ADEs, and PDEs dopaminergic neurons in young day 1 and old day 7 nematodes in both BY273 and *nth-1;*BY273 animals (n = 30 from three independent experiments; ^∗∗∗^p < 0.001; one-way ANOVA followed by Bonferroni’s multiple comparison test). (D) Survival of anterior CEPs and ADEs DA neurons of BY273 and *nth-1;* BY273 nematodes during aging (n = 35 nematodes per group; ^∗∗∗^p < 0.001; one-way ANOVA followed by Šidák’s multiple-comparisons test). Representative images of the head region of BY273 and *nth-1;* BY273 mutants at day 7 of adulthood. Remnants of neuronal cell bodies and axonal beading are scored in BY273 animals. Neuronal soma and processes architecture are maintained in NTH-1-deficient PD nematodes (scale bar, 50 μm). (E) The column scatterplots represent GFP intensity of the CEPs and ADEsand PDEs DA neurons in both BY273 and *nth-1;*BY273 animals in response to 6-OHDA (30 mM) and MPP^+^ (2 mM); n = 30 from three independent experiments; ^∗∗^p < 0.01, ^∗∗∗^p < 0.001; one-way ANOVA followed by Bonferroni’s multiple comparison test. The corresponding fluorescence image is depicted in Figure S1B. Error bars, SEM.

Base excision repair (BER) is the prominent pathway for repair of oxidative damage to DNA bases (Bosshard et al., 2012). This pathway is composed of a series of fine-tuned enzymatic steps; the first step is carried out by one of several substrate-selective DNA-glycosylases, which excise damaged DNA bases (e.g., 8-hydroxyguanine; 8-oxoG) (Figure 1A). Because BER prevents accumulation of oxidative DNA damage, it protects neurons from the harmful effects of cytotoxic or mutagenic DNA lesions. However, if the amount of DNA damage exceeds the capacity of BER, or if DNA lesions are inefficiently or incompletely repaired, active BER can generate toxic BER intermediates, such as single-stranded DNA breaks. Toxic BER intermediates also accumulate when the BER pathway becomes imbalanced, which can occur when BER enzymes are over- or under-expressed (Frosina, 2000).

Given the prominent role of oxidative stress in the etiology of PD and the importance of BER in neurons, we hypothesized that BER may serve as a major source of genomic stress in neurons. This hypothesis is supported by the following observations: first, the capacity to *initiate* BER in neurons is maintained or increased during aging, as indicated by upregulation of several DNA glycosylases in the substantia nigra (Bosshard et al., 2012; Fukae et al., 2005; Nakabeppu et al., 2007). Conversely, expression and activity of Polβ, the primary BER DNA polymerase, decline with age (Misiak et al., 2017; Sykora et al., 2015). Second, biochemical studies in mice indicate that the capacity to *complete* BER decreases with age (Cabelof et al., 2002a, 2002b; Rao et al., 2000). Nevertheless, it has been difficult to unequivocally demonstrate that BER affects aging or age-related neurodegeneration. Unlike other DNA repair pathways, BER can be initiated by a large number of DNA glycosylases in mammalian cells, some of which have overlapping substrate specificities. Thus, there is extensive redundancy in the enzymatic machinery that initiates BER, even though the core BER components are unique and essential enzymes.

Comprehensive understanding of the role of BER in neurodegeneration in mammals has been elusive, in part because mammalian cells express as many as 11 distinct DNA glycosylases with overlapping substrate specificities. The *Caenorhabditis elegans* genome possesses a simpler DNA glycosylase repertoire, encoding only two DNA glycosylases. The nematode enables *a priory* targeted genetic studies in BER initiation as redundancy is lower than in mammals. Therefore, *C. elegans* serves as an ideal model organism to systematically investigate whether incomplete or inefficient BER drives neuronal loss. *C. elegans* DNA glycosylases include UNG-1, a monofunctional DNA glycosylase that primarily excises uracil from DNA, leaving an apurinic/apyrimidinic site (AP site) (Skjeldam et al., 2010), and NTH-1, which excises oxidized DNA bases. NTH-1 is a bifunctional DNA glycosylase that excises damaged bases and also incises the DNA phosphodiester backbone 3′ to the AP site. This generates a nicked intermediate with 5′-phosphate and 3′-α,β-unsaturated aldehyde DNA termini (Fensgård et al., 2010; Kassahun et al., 2018; Morinaga et al., 2009). The 3′ blocking group must be removed to generate a 3′-OH DNA terminus in order for subsequent steps of BER to proceed (e.g., single nucleotide gap-filling DNA synthesis followed by ligation). In *C. elegans*, the 3′-OH group can be generated by AP-endonuclease APN-1 (Zakaria et al., 2010) or by EXO-3 (Shatilla et al., 2005). Processing of the blocked 3′ terminus constitutes the rate limiting step of BER (Sobol et al., 2000), and lack of co-ordination or inefficient processing may lead to accumulation of toxic single-stranded DNA (ssDNA) breaks with blocked 3′ termini (Figure 1A).

The ease of genetic manipulation coupled with availability of sophisticated behavioral assays has also made *C. elegans* a system of choice for unravelling evolutionarily conserved cellular and genetic pathways that regulate neuron development and function (Bhattacharya et al., 2019). *C. elegans* provides *in vivo* single neuron resolution over the entire natural lifespan of the organism. Moreover, well-characterized *C. elegans* PD models mimic the major features of the human PD pathology (Schmidt et al., 2007), including age-dependent loss of DA neurons accompanied by progressive neuro-motor dysfunction, such as bradykinesia and the inability to slow down or change direction in response to sensory input.

Here, we investigate the involvement of NTH-1 in neuronal survival using a well-established nematode model of PD. We found that incomplete repair of endogenous DNA base damage via NTH-1-initiated BER in both mitochondrial and nuclear DNA generates genomic stress during aging. This result suggests that imbalanced BER might be the driving force of neurodegeneration in our experimental model system. Furthermore, we observed that NTH-1 deficiency causes mitochondrial dysfunction and elicits an LMD-3/JNK-1/SKN-1-dependent mitohormetic response, which in turn protects DA neurons.

## Results

### NTH-1 deficiency attenuates α-synuclein neurotoxicity in DA neurons

In the PD nematode model (BY273), overexpression of α-synuclein (α-syn) in DA neurons triggers gradual neurodegeneration (Lakso et al., 2003). To generate a BER-defective PD model, we crossed the *nth-1(ok724)* loss of function allele (Fensgård et al., 2010; Kassahun et al., 2018) into the BY273 (*p*_*dat-1*_GFP; *p*_*dat-1*_WTα-syn) background (Nass and Blakely, 2003). While we expected, and did observe, progressive loss of DA neurons in aged BY273 animals, loss of DA neurons was not observed in NTH-1-deficient BY273 nematodes (Figures 1B and 1D), a result that was surprising and not expected. Whereas DA neuron viability, as measured by GFP intensity (Figures 1B and 1C) or by visual inspection (Figure 1D), was reduced more than 50% in 7-day-old relative to 1-day-old BY273 animals, DA neuron viability showed no decrease in 7-day-old *nth-1;*BY273 animals (Figure 1B). The apparent neuroprotective effect of NTH-1 deficiency was not due to altered transcriptional activity of the tissue-specific *dat-1* promoter (Figure S1A).

Several chemical compounds, including known neurotoxins and insecticides, are associated with increased risk of developing parkinsonism (Nass et al., 2002). To confirm that loss of NTH-1 reduced vulnerability of DA neurons to chemically induced damage and decreased viability, we measured survival of DA neurons in the absence or presence of the dopamine analog 6-hydroxy dopamine (6-OHDA) or the mitochondrial poison 1-methyl-4-phenylpyridinium (MPP+). Both of these neurotoxins generate ROS by inhibiting flux through the mitochondrial electron transport chain, resulting in ROS-induced neuronal loss (Hernandez-Baltazar et al., 2017; Rossetti et al., 1988). The results show that DA neurons in *nth-1;*BY273 mutants were more resistant to 6-OHDA- and MPP^+^-induced neurotoxicity than DA neurons in BY273 animals (Figure 1E; Figure S1B). These results suggest that NTH-1 deficiency protects against age- and oxidative-stress-related degeneration of DA neurons.

For comparison, similar experiments were performed in nematodes carrying a null mutation in the gene encoding *C. elegans* DNA glycosylase UNG-1 (Skjeldam et al., 2010). Although UNG-1-deficient nematodes showed slightly higher survival of DA neurons than wild-type animals, UNG-1 deficiency conferred a substantially weaker neuroprotective effect than NTH-1 deficiency (Figure S1C). Thus, neuronal homeostasis depends highly on NTH-1 activity. Moreover, we examined the impact of depleting additional BER enzymes APN-1 and EXO-3 on the survival of DA neurons in BY273 and *nth-1*;BY273 nematodes. Depletion of APN-1 or EXO-3 provided no neuroprotection in BY273 nematodes (Figure S1D), while, intriguingly, APN-1 was required for neuroprotection in *nth-1*;BY273 animals, and EXO-3 was not (Figure S1D).

To investigate whether defects in other DNA repair pathways also promote DA survival, we knocked down, pan-neuronally, several components of the nucleotide excision repair (NER) and non-homologous end joining (NHEJ) pathways in BY273 and *nth-1*;BY273 animals. In contrast to the neuroprotective effect observed in NTH-1-deficient animals, depletion of CSA-1, CSB-1, or XPA-1 increased DA neuronal loss in BY273 and *nth-1*;BY273 nematodes (Figures S2A–S2C), which is consistent with previously reported studies in mammalian cells (Hou et al., 2019). Interestingly, depletion of LIG-4 and CKU-80 resulted in moderate reversal of DA neuron survival in *nth-1*;BY273 nematodes. These results suggests some involvement of NHEJ in the neuroprotective effect of NTH-1 deficiency (Figures S2B and S2C).

### NTH-1 loss improves dopamine-dependent behavior

**Figure 2:**
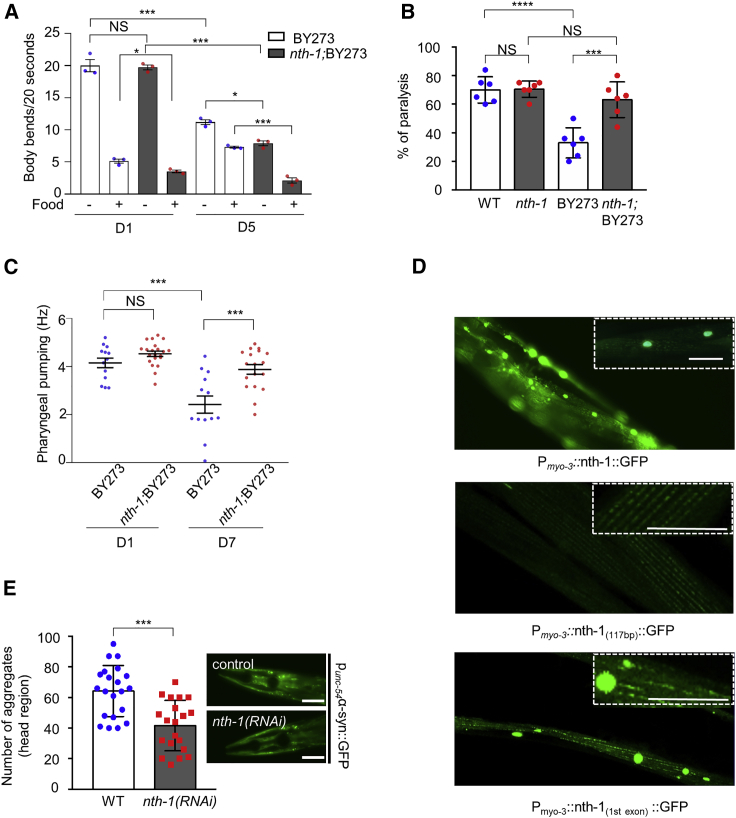
Loss of NTH-1 improves neuronal function and homeostasis (A) Basal slowing response of nematodes co-expressing human α-synuclein (α-syn) in the transgenic strain BY273 and *nth-1;*BY273. Body bends per 20 s measured on NGM plates with and without bacteria. n = 30 individuals for each strain were scored in three independent experiments. The columns represent mean, and the scatterplot represents mean of each experiment; NS p > 0.05, ^∗^p > 0.05, ^∗∗∗^p < 0.001; one-way ANOVA followed by Bonferroni’s multiple comparison test. (B) Transgenic animals expressing human α-syn in DA neurons are less sensitive to dopamine-induced paralysis (40 mM). NTH-1 deficiency abolishes dopamine resistance (n = 40 nematodes per group; ^∗∗∗∗^p < 0.0001, ^∗∗^p < 0.001; one-way ANOVA). (C)The dot plots represent the quantification of pharyngeal pumping frequency of day 1 and day 7 adults in transgenic strain BY273 and *nth-1;*BY273 (n = 15–20 individuals; NS p > 0.05, ^∗∗∗^p < 0.001; one-way ANOVA followed by Bonferroni’s multiple comparison test). (D) In the top panel, transgenic animals expressing NTH-1 fused with GFP in body-wall muscle cells. NTH-1 displays both nuclear and mitochondrial localization pattern. Scale bar, 20 μm, 20× objective. In the middle panel, transgenic animals expressing the first 117bp of *nth-1* coding sequence fused with GFP in body-wall muscle. NTH-1 displays mostly a mitochondrial localization pattern. In the bottom panel, transgenic animals expressing the first exon of NTH-1 fused with GFP in body-wall muscle. NTH-1 displays both mitochondrial and nuclear localization pattern. Scale bar, 20 μm, 60× objective. (E) Transgenic nematodes expressing human α-synuclein fused with GFP in body wall muscle cells were subjected to RNAi against *nth-1.* α-syn aggregates are decreased following knocking down of *nth-1* (n = 20 nematodes per group; ^∗∗∗^p < 0.001; unpaired t test). Representative images of the head region of WT animals and NTH-1-depleted animals at day 5 of adulthood. Scale bar, 20μm, 20× objective. Error bars, SEM.

The BY273 PD nematode model exhibits several dopamine-dependent phenotypic abnormalities (Cooper and Van Raamsdonk, 2018). To assess whether NTH-1-deficient animals demonstrate both neuroprotection and improved dopamine-dependent neuronal function, we measured the basal slowing response, a standard method for evaluating dopaminergic signaling. The basal slowing response measures the rate of locomotion of well-fed nematodes when transferred to solid media with or without a bacterial lawn (Sawin et al., 2000). In young *nth-1;*BY273 and BY273 nematodes, there was no difference in the number of body bends in the absence of food, nor in the basal slowing ratio in the presence of food, indicating that NTH-1-deficient animals do not display any intrinsic movement deficit (Figure 2A). In 5-day-old adults, movement was slower for both BY273 and *nth-1*;BY273 nematodes in the absence of food as expected, but the basal slowing ratio in the presence of food was more significant for *nth-1*;BY273 than for BY273 animals (Figure 2A), a result that suggests improved response to dopamine and improved neuronal function. Confirming this observation, *nth-1;*BY273 nematodes demonstrated a lower percentage of paralysis in response to exogenous dopamine (Figure 2B) and 5-day-old adults produced viable broods, while 5-day-old BY273 nematodes did not (Figure S2D). Moreover, aged *nth-1;*BY273 mutants retained high pharyngeal pumping capacity (Figure 2C), a commonly used health-span marker (Fang et al., 2017). Taken together, our results show that loss of NTH-1 improves general health of aging animals in addition to reducing age-related loss of DA neurons in PD nematodes.

### Hormesis promotes neuroprotection against α-synuclein in *nth-1* mutants

To gain insight into the molecular mechanisms that improve the survival of DA neurons, we examined stress response pathways commonly involved in preserving cellular functionality. Much of this work was conducted in *nth-1* mutants (non-PD model), which display mild mitochondrial dysfunction, as described in greater detail elsewhere (Kassahun et al., 2018) and in Figure S3. The mild mitochondrial dysfunction may be a direct consequence of defects in mitochondrial DNA repair, as NTH-1 is active in both the nucleus and in mitochondria (Figure 2D). Some nuclear and mitochondrial localization was achieved with the 39 N-terminal amino acids of NTH-1 (Figure 2D, middle panel), but the entire exon 1 was required for efficient targeting to both compartments (Figure 2D, bottom panel). The mild mitochondrial dysfunction may therefore be a direct consequence of defects in mitochondrial DNA repair, which is supported by moderate mitochondrial fragmentation observed in the intestinal and body-wall muscle cells of *nth-1* animals (Figures S3A–S3F). While the mitochondrial mass was unaltered in intestinal and body-wall muscle cells, the number of mitochondria was lower in axons of *nth-1* mutants than in control animals (Figures S3G and S3H), suggesting that neuronal mitochondria may be especially vulnerable to loss of NTH-1. We hypothesized that one mechanism by which NTH-1 deficiency might confer neuroprotection would be through inhibition of α-syn aggregate formation, a cardinal feature of PD pathology. In fact, knocking down NTH-1 in transgenic nematodes expressing α-syn fused with GFP in body-wall muscle cells resulted in fewer α-syn aggregates than in control animals. This could reflect decreased formation or increased clearance of α-syn aggregates in these animals (Figure 2E).

Because autophagy is a cellular catabolic process that mediates the clearance of protein aggregates and damaged organelles (Leidal et al., 2018; Schiavi and Ventura, 2014), the efficiency of autophagy could also have an impact on α-syn aggregation in PD. Therefore, we assessed the number of autophagosomes in *nth-1* mutants by monitoring expression of the autophagosomal protein LGG-1 fused either with GFP or DsRed in several neuronal cell types. Notably, based on these markers, neuronal autophagy was not induced in *nth-1* mutants (Figures S4A and S4B). Moreover, NTH-1 depletion did not alter formation of autophagosomes in intestinal or body wall muscle cells (Figures S4C and S4D), nor trigger nuclearization of HLH-30 (homolog of the mammalian TFEB), a master transcriptional regulator of genes involved in lysosome biogenesis and autophagy (Figure S4E). Consistently, genetic ablation of *lgg-2* in *nth-1*;BY273 nematodes did not alter the viability of DA neurons (Figure S4F). These data indicate that neuroprotection conferred by NTH-1 deficiency in the nematode PD model is not dependent on autophagy. Further, mitophagy was not activated in *nth-1* mutants expressing a mitochondria-targeted Rosella biosensor in neurons (Fang et al., 2019; Palikaras et al., 2015) (Figures S4G and S4H). Last, knockdown of NTH-1 did not stimulate expression of *hsp-60*, indicating that the mitochondrial unfolded protein response (UPR^mt^) was not induced in NTH-1-depleted cells (Figure S4I).

**Figure 3:**
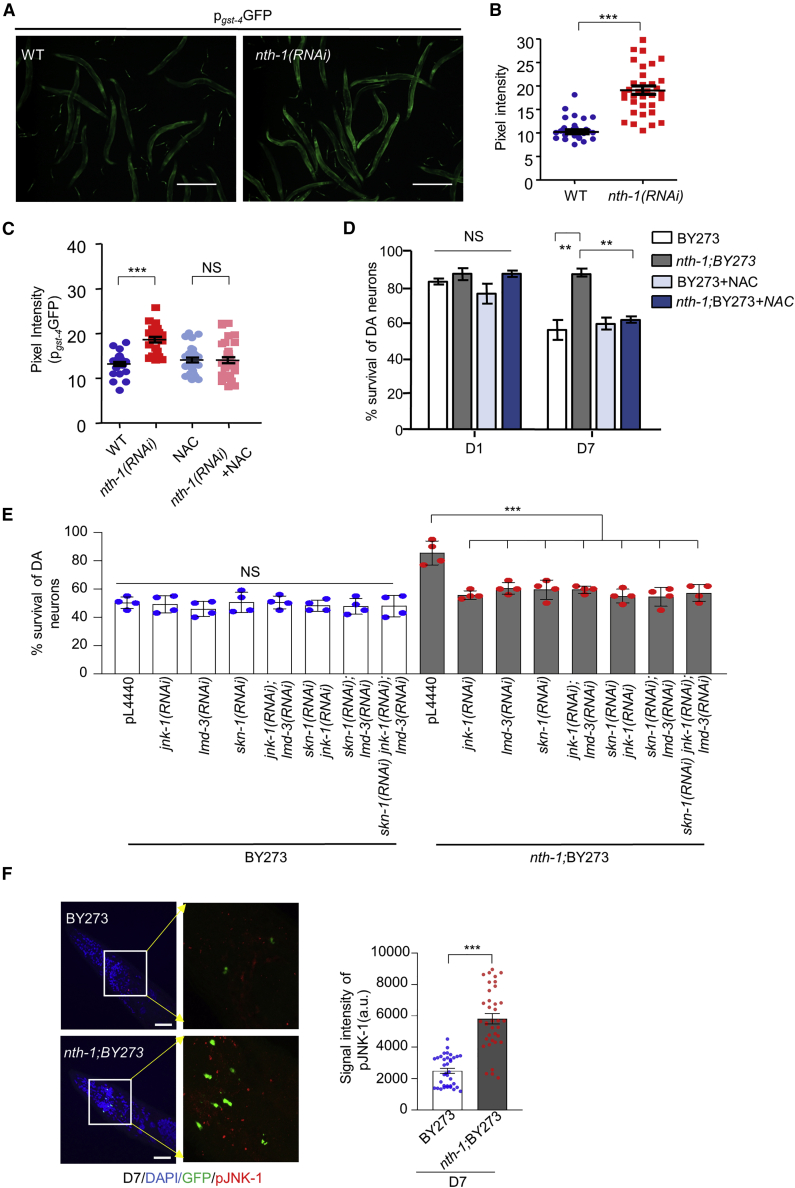
NTH-1 deficiency initiates a mitohormetic response that promotes neuroprotection (A and B) SKN-1 is activated in NTH-1-depleted animals. Fluorescence intensity of transgenic animals expressing the p_*gst-4*_GFP transgene subjected to *nth-1* knockdown (n = 45; ^∗∗∗^p < 0.001; unpaired t test). Scale bar, 500 μm, 5× objective. (C) SKN-1 is not stimulated in NTH-1 knocked down nematodes upon NAC administration (n = 45; NS p > 0.05, ^∗∗∗^p < 0.001; one-way ANOVA). (D) Mitohormesis is engaged in NTH-1-deficient animals to induce neuroprotection. Supplementation of NAC ameliorates the neuroprotective effect of NTH-1 deficiency (n = 40; ^∗∗^p < 0.01; one-way ANOVA), corresponding image in Figure S5C. (E) The column scatterplots represent survival of CEPs and ADEs DA neurons of BY273 and *nth-1;*BY273 nematodes during aging following knockdown of *jnk-1*, *lmd-3*, and *skn-1* and co-depletion of *jnk-1/lmd-3*, *skn-1/jnk-1*, *and skn-1/lmd-3* and *simultaneous* depletion of *skn-1*/*jnk-1*/*lmd-3* by RNAi (n = 40–55 nematodes per group; ^∗∗^p < 0.01 and ^∗∗∗^p < 0.001; one-way ANOVA followed by Bonferroni’s multiple comparison test), corresponding image Figure S5E. (F) Immunofluorescence images showing anti-pJNK-1 positive foci in the head region of transgenic strains BY273 and *nth-1;*BY273 (scale bar, 20 μm, zoomed, 5 μm, 63× objective). The column scatterplot represents the average signal intensity of pJNK-1 staining in a 17 × 10^8^ nm^2^ area in the head region of transgenic animals (right panel; n = 12 animals per replicate, three replicates; ^∗∗∗^p < 0.001; two-way ANOVA. Error bars, SEM.

Given that our prior studies had demonstrated that *nth-1* mutant animals exhibit moderately elevated levels of ROS due to perturbations in mitochondrial function (Kassahun et al., 2018), and rewiring of transcription programs (Fensgård et al., 2010) involving upregulation of stress response genes and downregulation of some genes consistent with suppression of insulin like signaling (Tables S1 and S2), we explored whether NTH-1 deficiency might promote an adaptive, cytoprotective response, known as mitohormesis (Blackwell et al., 2015; Palmeira et al., 2019; Ristow, 2014; Ristow and Zarse, 2010). Recent studies show that transcription promoted by SKN-1, a master transcriptional regulator of the oxidative stress response, promotes mitochondrial homeostasis and mitohormesis-mediated longevity (Palikaras et al., 2015; Pinto et al., 2018; Schmeisser et al., 2013; Zarse et al., 2012). SKN-1 activity increased in animals exposed to *nth-1* RNAi, as was evident by elevated expression of a GFP transgene under the control of the *gst-4* promoter, a well-known SKN-1 target gene (Figures 3A and 3B; Kassahun et al., 2018). If SKN-1 is activated in response to high levels of ROS, we would expect that ROS scavengers would suppress its activation. Indeed, when N-acetyl-L-cysteine (NAC) was added to growth medium, SKN-1 was not induced following RNAi knockdown of *nth-1* (Figure 3C). Although NAC did not prevent death of DA neurons in the nematode PD model, it abolished neuroprotection in *nth-1*;BY273 mutants (Figure 3D; Figures S5A–S5C). These findings suggest that ROS-induced activation of SKN-1 is involved in the mechanism by which NTH-1 deficiency confers neuroprotection in the nematode PD model. Consistently, RNAi knockdown of SKN-1 attenuated neuroprotection in *nth-1*;BY273 nematodes (Figure 3E; Figure S5E).

Mitogen-activated protein kinases, such as Jun-N-terminal kinase (JNK-1), are potent regulators of cellular stress responses in *C. elegans* (Andrusiak and Jin, 2016). We previously found that PMK-1 and JNK-1 are constitutively active in the germline of *nth-1* mutants (Kassahun et al., 2018). However, depletion of *pmk-1* had no effect on survival of DA neurons (Figure S5D). Phosphorylated JNK-1 (pJNK-1) was also detected in the head-region of BY273 and *nth-1*;BY273 animals, but at a higher level in *nth-1*;BY273 nematodes (Figure 3F). Therefore, we examined whether JNK-1 activation was required for neuroprotection in *nth-1*;BY273 mutants. Notably, *jnk-1* RNAi abolished neuroprotection in *nth-1;*BY273 animals, while it modestly increased survival of DA neurons in BY273 nematodes (Figure 3E; Figure S5E). Simultaneous RNAi-mediated depletion of *skn-1* and *jnk-1* did not increase survival of DA neurons in *nth-1*;BY273 worms. These results indicate that JNK-1 is epistatic with SKN-1 to alleviate α-syn-induced neurotoxicity (Figure 3E; Figure S5E).

Oxidation resistance gene 1 (OXR1) is a conserved protein important for the response to oxidative stress (Elliott and Volkert, 2004; Yang et al., 2014). Recent studies show that OXR1 overexpression normalizes several pathological features in a mouse model of PD (Jiang et al., 2019). Consistent with this finding, we observed that LMD-3 (the *C. elegans* homolog of OXR1) is required for neuroprotection in *nth-1*;BY273 worms (Figure 3E; Figure S5E). Interestingly, a similar level of neuroprotection was observed after depletion of LMD-3 with or without depletion of JNK-1 and/or SKN-1. These findings suggest that LMD-3, JNK-1, and SKN-1 function in the same genetic pathway promoting neuronal survival (Figure 3E; Figure S5E).

### Superoxide dismutases are required for neuroprotection in NTH-1-deficient animals

**Figure 4:**
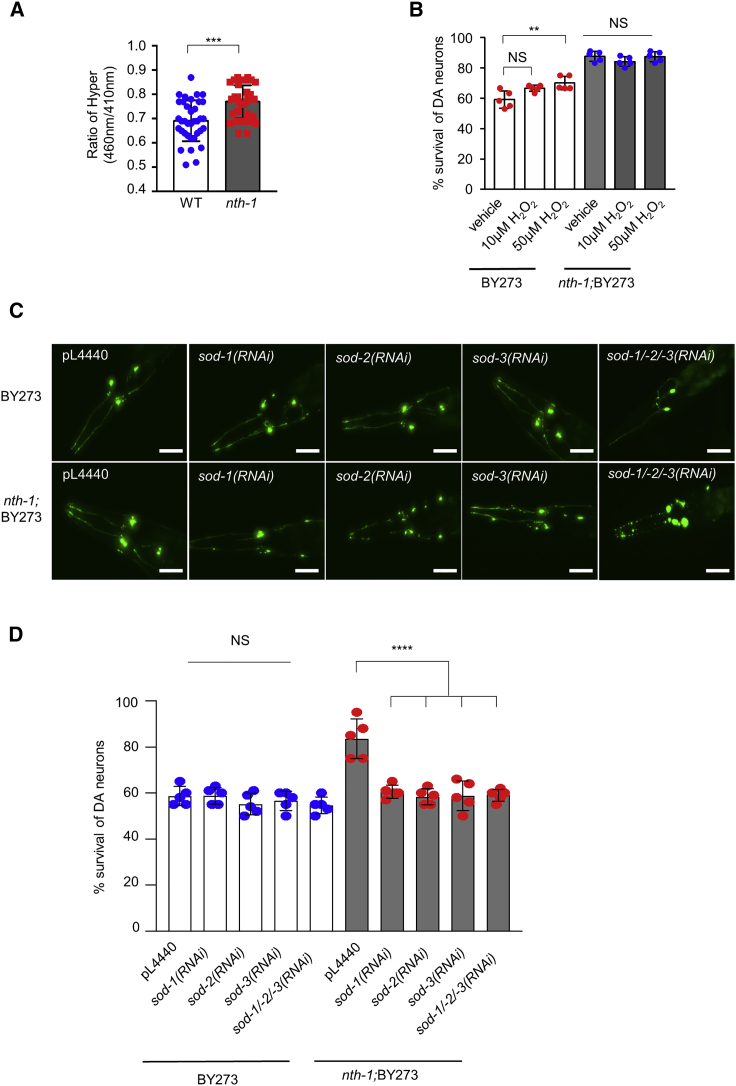
Hydrogen peroxide mediates the neuroprotective effect of NTH-1 deficiency in the nematode PD model (A) The cellular levels of hydrogen peroxide are elevated in NTH-1-depleted animals (n = 45; ^∗∗∗^p < 0.001; unpaired t test). (B) Exogenous supplementation of hydrogen peroxide (10 and 50 μM) prevents α-syn-induced neurodegeneration promoting a mitohormetic response (n = 40 nematodes per group; NS p > 0.05 and ^∗∗^p < 0.01; one-way ANOVA). (C and D) Knockdown of SOD-1, SOD-2, SOD-3, and simultaneous depletion of SOD-1/SOD-2/SOD-3 superoxide dismutases abolishes the neuroprotective effect of NTH-1 deficiency in PD nematodes (n = 40 nematodes per group; ΝS p > 0.05 and ^∗∗∗∗^p < 0.0001; one-way ANOVA). Scale bar, 20 μm, 20× objective. Error bars, SEM.

The above data indicate that NTH-1-deficient animals experience chronic oxidative stress, which activates cellular pathways that prevent oxidative stress-induced neuronal degeneration. Hydrogen peroxide is a redox-signaling molecule, generated as a byproduct of cellular energy metabolism, that modulates cellular stress responses (Sies, 2017). To provide further mechanistic insights and to confirm the hormetic response in *nth-1* mutants, we monitored the intracellular levels of hydrogen peroxide. We used transgenic animals expressing the Hyper biosensor (Back et al., 2012; Knoefler et al., 2012) and found increased levels of hydrogen peroxide in *nth-1*;BY273 animals (Figure 4A). To confirm that hydrogen peroxide acts as a signaling molecule to promote neuroprotection in PD animals, we exposed nematodes to low doses of hydrogen peroxide to trigger hormesis (Bhatla and Horvitz, 2015). Indeed, addition of 50 μΜ hydrogen peroxide to the culture medium enhanced the survival of DA neurons in BY273 worms (Figure 4B), supporting the idea that hydrogen peroxide plays a direct role in protecting DA neurons against α-syn-induced neurodegeneration. Interestingly, hydrogen peroxide did not increase survival of DA neurons in *nth-1;*BY273 animals, suggesting that the basal activation of the oxidative stress response is sufficient to promote neuronal survival in this genetic background.

To further investigate the molecular mechanism underlying protection of DA neurons in *nth-1* mutants, we assessed the requirement for superoxide dismutases (SODs), which generate hydrogen peroxide from superoxide anion (Wang et al., 2018b). The *C. elegans* genome contains five genes that encode SODs, and SKN-1 has been implicated in transcriptional regulation of three of these genes, *sod-1*, *sod-2*, and *sod-3* (An and Blackwell, 2003). We examined whether these three enzymes are required for survival of DA neurons in *nth-1* PD nematodes by knocking down *sod-1*, *sod-2*, or *sod-3* individually or simultaneously. Interestingly, depletion of SOD-1/SOD-2/SOD-3 abolished the neuroprotective effect of *nth-1*;BY273 nematodes but did not influence survival of BY273 animals (Figures 4C and 4D). To investigate the prominent contribution of specific tissues to the neuroprotective effect of NTH-1 deficiency, we targeted the expression of *sod-1*, *sod-2*, and *sod-3* in the hypodermis and intestine. We found that hypodermal or intestinal knockdown of SOD-1, SOD-2, and SOD-3 did not increased survival of DA neurons in *nth-1*;BY273 animals. These results demonstrated that SODs promote neuroprotection in a neuron-specific manner (Figure S5F). Elaborating further on the cell-autonomous function of NTH-1 in the tissues of *C. elegans*, we found that pan-neuronal or DA neuron-specific knockdown of NTH-1 promoted neuronal viability (Figures S6A–S6D), whereas hypodermal or intestinal RNAi against *nth-1* did not provide any neuroprotective effect (Figures S6E–S6H). Taken together, our analysis reveals that neuronal loss of NTH-1 increases production of hydrogen peroxide in an SOD-dependent manner, which in turn activates an LMD-3/JNK-1/SKN-1-dependent signaling pathway promoting survival of DA neurons in *nth-1*;BY273 nematodes. Collectively, the above results strongly support a mechanistic link between moderate mitochondrial impairment, ROS-mediated signaling, and neuroprotection in *nth-1*;BY273 mutants.

### NTH-1 deficiency diminishes age-dependent accumulation of single-stranded DNA breaks

**Figure 5:**
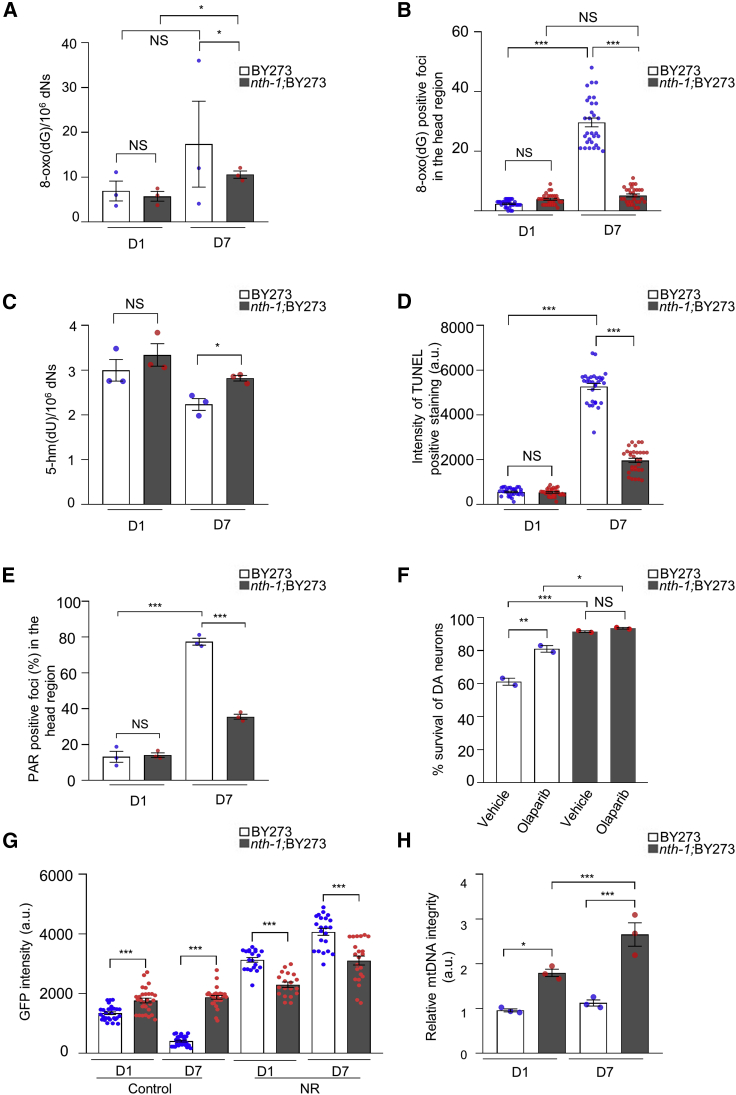
NTH-1 deficiency diminishes age-dependent genomic damage (A) Quantification of 8-hydroxy deoxyguanosine (8-oxoG) level in genomic DNA of BY273 and *nth-1;* BY273 animals are represented as a column scatterplots (n > 1,000 nematodes per group; three replicates; NS p > 0.05 and ^∗^p < 0.05; unpaired t test). (B) The scatter dot plot shows the number of 8-oxoG positive foci in the head region of transgenic animals (n = 30 nematodes; NS p > 0.05, ^∗∗∗^p < 0.001; one-way ANOVA followed Bonferroni’s multiple comparison test). The corresponding image is depicted in Figure S7A. (C) Quantification of 5-hmU levels in genomic DNA of BY273 and *nth-1;*BY273 animals is presented as a column scatterplot (n > 1,000 nematodes per group; three replicates; NS p > 0.05, ^∗^p < 0.05; unpaired t test). (D) Scatter dot plot showing the intensity of TUNEL positive staining in 17 × 10^8^ nm^2^ area in the head region of transgenic animals (n = 30 nematodes; ^∗∗∗^p < 0.001; one-way ANOVA followed by Bonferroni’s multiple comparison test). The corresponding image is depicted in Figure S7B. (E) Quantification of the fraction (%) of heads with PAR positive foci (n = 12–24 nematodes per group; three replicates; NS p > 0.05, ^∗∗∗^p < 0.001; one-way ANOVA followed by Bonferroni’s multiple comparison test). The corresponding image is shown in Figure S7D. (F) The column scatterplot depicts survival of anterior CEPs and ADEs DA neurons of BY273 and *nth-1;*BY273 nematodes during aging in response to 0,5 μM olaparib (n = 30 to 40 animals per replicate, two replicates; NS p > 0.05, ^∗^p < 0.05, ^∗∗^p < 0.01,^∗∗∗^p < 0.001; one-way ANOVA followed by Bonferroni’s multiple comparison test). (G) Column scatterplot representing survival of anterior CEPs DA neurons (measured as mean GFP intensity) of BY273 and *nth-1;*BY273 nematodes during aging with and without 1 mM NR (n = 10 to 30 animals per replicate, two replicates, ^∗∗∗^p < 0.001; one-way ANOVA followed by Bonferroni’s multiple comparison test). (H) Quantification of relative mtDNA integrity in N2 and *nth-1* mutants in young and old animals (n = 800 animals per replicate, three replicates; ^∗^p < 0.05 and ^∗∗∗^p < 0.001; one-way ANOVA followed by Bonferroni’s multiple comparison test). Error bars, SEM.

To further delineate the molecular mechanism connecting BER deficiency and survival of DA neurons in PD nematodes, we examined whether neuroprotection was associated with a reduction in DNA damage. Preferred substrates of NTH-1 are oxidized pyrimidines (for example, 5-hydroxymethyluracil [5-hmU]), but a minor activity was reported toward 8-hydroxyguanine (8-oxoG) in an unusual base pair context (i.e., 8-oxoG:G) (Morinaga et al., 2009). We measured the levels of oxidized DNA bases in wild-type and NTH-1-deficient PD nematodes during aging by liquid chromatography-tandem mass spectrometry (LC-MS/MS) (Figure 5A) and by immunohistochemical staining with anti-8-oxoG antibodies (Figure 5B; Figure S7A). The results show that young *nth-1;*BY273 mutants had similar levels of 8-oxoG as isogenic BY273 nematodes and that the level of 8-oxoG increased in NTH-1-deficient and NTH-1-proficient nematodes with age (Figures 5A and 5B; Figure S7A). However, the age-dependent increase in 8-oxoG (positive foci in the head region) was more pronounced in the BY273 animals than in *nth-1*;BY273 mutants, consistent with upregulation of antioxidant defense systems in the latter background (Figure 3; Figure S5). With regard to one of the preferred substrates of NTH-1, 5-hmU, a small increase in the level of 5-hmU was observed in older *nth-1;*BY273 mutants relative to the BY273 nematodes (Figure 5C), showing that the boosted antioxidant defense is not sufficient to prevent accumulation of oxidized pyrimidines. We conclude that the increased level of 5-hmU is likely a direct consequence of *nth-1* deletion, whereas the change in level of 8-oxoG is likely an indirect effect of mitohormesis.

To determine whether the reduced age-related degeneration of DA neurons in *nth-1* mutants was associated with lower levels of cytotoxic DNA repair intermediates, we measured the abundance of ssDNA breaks using the TUNEL assay. Although commonly used as an assay for apoptosis, the TUNEL assay quantifies incorporation of labeled dUTP at 3′-OH single-stranded DNA ends, which can be generated during BER by the combined action of NTH-1 and AP-endonuclease. TUNEL data revealed a barely detectable level of ssDNA breaks in young animals of either genotype (Figure 5D). However, older BY273 animals displayed a substantial increase in the number of TUNEL-positive cells in the head region, while a smaller modest increase in TUNEL staining was observed in older *nth-1;*BY273 worms (Figure 5D; Figure S7B). Annexin V staining was not detected in the head region of older BY273 animals, which supports the idea that the TUNEL signal represents 3′-OH single-stranded DNA breaks generated by NTH-1 initiated BER (Figure S7C). Furthermore, anti-poly(ADP-ribose) (PAR) positive foci, which are generated at ssDNA breaks by poly (ADP-ribose) polymerase 1 (PARP1), were observed in nearly 80% of older BY273 animals and in <60% of older *nth-1;*BY273 animals (Figure 5E; Figure S7D). These data suggest that NTH-1-dependent BER generates ssDNA breaks in aged PD nematodes.

Unlike most base adducts, ssDNA breaks are cytotoxic lesions that can induce neurodegeneration through direct signaling to classic apoptotic pathways (Hanzlikova et al., 2018) or, indirectly, by activating a cascade of events leading to depletion of NAD^+^ (Fang et al., 2014, 2016). Supporting the idea that BER-generated ssDNA breaks can lead directly to neurotoxicity, we observed that inhibition of PARP1 with Olaparib improved survival of DA neurons in BY273 animals, while it did not improve survival of DA neurons in *nth-1*;BY273 nematodes (Figure 5F; Figure S7E). Consistently, boosting NAD^+^ levels by supplementation with nicotinamide riboside (NR) improved survival of DA neurons in BY273 animals substantially, whereas the benefit was less pronounced in *nth-1;*BY273 mutants (Figures 1C and 5G; Figure S7F). Taken together, these data suggest that repair of endogenous DNA damage via BER generates a genomic stress signal that drives degeneration of DA neurons during aging of BY273 nematodes.

Recent studies demonstrate a strong relationship between nuclear genome maintenance and energy homeostasis (Fang et al., 2014, 2016). Therefore, we explored whether nuclear NTH-1 deficiency had an impact on energy homeostasis in BY273 nematodes via nuclear mitochondrial signaling. Due to NTH-1 mitochondrial localization (Figure 2D), we explored whether mitohormesis could be caused by defective mitochondrial BER. Measurements of DNA lesions in the total genomes of NTH-1-deficient and NTH-1-proficient BY273 animals (Figure 5C) suggest that bona fide NTH-1 substrates most likely accumulate in mtDNA of *nth-1* mutants. However, it is currently not possible to isolate enough mtDNA and measure lesions directly by LC-MS/MS. To confirm that incomplete NTH-1-initiated BER also results in mtDNA intermediates, we therefore assessed mtDNA integrity using a PCR-based method, where damage that negatively affect PCR efficiency, such as single-strand breaks and cytotoxic base damage, is reflected by reduced PCR efficiency of the isolated template (Gonzalez-Hunt et al., 2016). Young *nth-1*;BY273 mutants had higher mtDNA integrity than BY273 animals and this effect was even more pronounced in old *nth-1;*BY273 nematodes (Figure 5H). Thus, we conclude that NTH-1 generates DNA lesions (likely ssDNA breaks) in the nuclear and mitochondrial genomes. Hence, the neuroprotective effect of NTH-1 deficiency in old *nth-1;*BY273 animals could directly reflect fewer ssDNA breaks and a higher proportion of high-integrity mtDNA.

### NTH-1 deficiency elevates age-dependent mitochondrial gene expression

**Figure 6:**
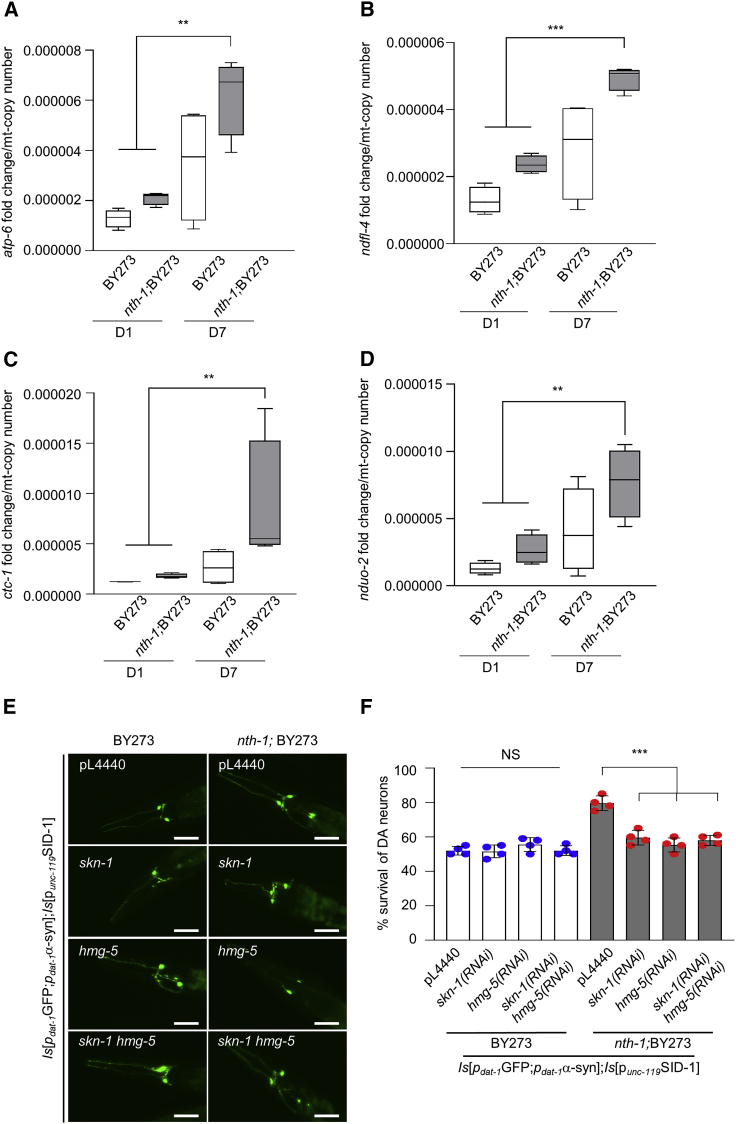
Mitohormesis in NTH-1-deficient animals depends on transcription (A–D) The boxplot with whiskers represents the expression of mitochondrial-specific gene *atp-6*, *ndfl-4*, ctc-*1*, and *nduo-2* expression normalized to mitochondrial copy number (n = 8 animals per group, three biological replicates; ^∗∗^p < 0.01, ^∗∗∗^p < 0.001; one-way ANOVA followed by Tukey’s multiple comparison test). (E) Representative images of the head region of transgenic animals BY273 and *nth-1;*BY273 at day 7 of adulthood after knockdown of *hmg-5*, *skn-1*, and co-knockdown of *skn-1* and *hmg-5* (Scale bar, 20 μm, 20× objective len). (F) The column scatterplots represent survival of anterior CEPs and ADEs DA neurons of BY273 and *nth-1;*BY273 nematodes upon knockdown of *hmg-5*, *skn-1*, compared with animals subjected to simultaneous knockdown *of hmg-5 and skn-1* (n = 40–55 nematodes per group; NS p > 0.05 and ^∗∗∗^p < 0.001; one-way ANOVA followed Bonferroni’s multiple comparison test). Error bars, SEM.

Transcription stress is believed to be an important contributor to accelerated aging in many DNA repair mutants. Older *nth-1*;BY273 worms with higher mtDNA integrity are expected to have higher levels of oxidized DNA bases on a genome-wide level (Figure 5C), and these lesions could potentially reduce the efficiency of transcription or modulate its regulation (Bordin et al., 2021). To clarify the apparent paradox between mtDNA integrity and mild mitochondrial dysfunction, we measured the steady-state levels of all mitochondrially encoded transcripts in single worms. Levels of mitochondrially encoded transcripts, adjusted for mitochondrial copy number, increased in old animals compared to young animals. Interestingly, *nth-1;*BY273 nematodes expressed dramatically higher levels of transcripts from select mitochondrial genes *atp-6*, *ndfl-4*, *ctc-1*, and *nduo-2* than BY273 worms (Figures 6A–6D). This change was consistent also in old NTH-1 mutant only as compared to wild-type N2 (Figures S8B–S8E). As the mitochondrial copy number did not increase systemically (Figures S3A–S3F; Figure S8A) and, in fact, showed a small decrease in axons (Figures S3G and S3H) in *nth-1*;BY273 (relative to BY273 animals), the increase in transcription could not be ascribed to increased mtDNA copy number (Figure S8A). Thus, it appears that loss of NTH-1 results in a dramatic accumulation of mtDNA-encoded transcripts with age.

The increased steady-state levels of a large fraction of mitochondrially encoded transcripts are reminiscent of a state of hypertranscription, which is emerging as a powerful inducer of nuclear stress response pathways (Kotsantis et al., 2016). Thus, we asked whether transcription was required for induction of mitohormesis in *nth-1*-deficient animals. To address this question, we examined the role of HMG-5 (homolog of the mammalian mitochondrial transcription factor A; TFAM) that is the master regulator of mtDNA transcription and replication. Although downregulation of *hmg-5* using RNAi did not affect the survival of DA neurons in old BY273 animals (Figures 6E and 6F), survival of DA neurons in old *nth-1*;BY273 nematodes decreased significantly, with or without simultaneous depletion of SKN-1 (Figures 6E and 6F). To confirm the cell-autonomous function of NTH-1, SKN-1, and HMG-5, we targeted their gene expression in DA neurons. Although, NTH-1 DA-neuron-specific depletion promoted neuronal survival as expected, simultaneous knockdown of *hmg-5* and *skn-1* abolished the neuroprotection conferred by NTH-1 knockdown. HMG-5 is also required for mtDNA replication, but given the dramatic change in transcription output and the very small change in mtDNA copy number in the *nth-*1 background, our data suggest that mitohormesis in response to *nth-1* depletion depends on mtDNA transcription.

### BER is a susceptibility modifier in PD

To explore whether BER might influence the risk of developing PD in humans, we analyzed whole-exome sequencing data from two independent cohorts of patients with idiopathic PD and healthy controls (Gaare et al., 2018). Through pathway-based enrichment analyses for rare nonsynonymous, stopgain, stoploss, and splice variants using the SKAT-O test, a significant enrichment of variants in BER genes was found (SKAT-O: p = 0.03). The effect was driven by *NEIL2*, a DNA glycosylase having substrate specificity similar to that NTH-1 (Table S3).

Gene-level analyses revealed significant enrichment (p = 0.049, after Bonferroni correction) of a single variant, (rs150931138), Gly26Ala NEIL2. This variant was detected in 6/411 PD cases and 0/640 controls (Table S3). Thus, genetic variation in genes in the BER pathway may contribute to susceptibility of PD in humans, but individual genetic variants of DNA glycosylase genes are expected to have a modest effect on PD risk.

## Discussion

**Figure 7:**
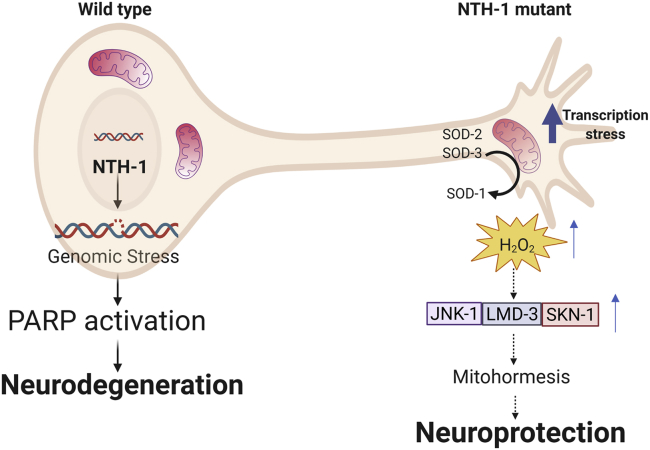
BER act as a susceptibility modifier in *C. elegans* PD animals Age-dependent accumulation of mitochondrial and nuclear DNA repair intermediates are generated through incomplete repair of endogenous base damage via the BER pathway. These repair intermediates promote DA neuronal vulnerability and degeneration in wild-type PD animals. In NTH-1-deficient nematodes, BER generated repair intermediates are not generated, but a state of mild mitochondrial dysfunction is induced, resulting in elevated H_2_O_2_ levels through the activity of SODs. In turn, SKN-1, LMD-3, and JNK-1 are stimulated to orchestrate a response that protects DA neurons from α-syn induced neurotoxicity via mitohormesis.

Aging is considered the single most important risk factor for the development and progression of PD. Therefore, sporadic PD can be viewed as an accelerated form of normal aging, characterized by defective mitochondrial metabolism leading to premature death of DA neurons (López-Otín et al., 2013; Mullin and Schapira, 2013). Using *C. elegans* as a model organism, we demonstrate that NTH-1 initiated BER promotes accumulation of mitochondrial and nuclear DNA repair intermediates with age, which directly promote neurodegeneration (Figure 7). In NTH-1-deficient PD nematodes, the accumulation of DNA repair intermediates is attenuated. In addition, neuronal survival and activity is promoted by the mild mitochondrial dysfunction phenotype of *nth-1* mutants, which stimulates an LMD-3/JNK-1/SKN-1-dependent mitohormetic response associated with increased mitochondrial transcription.

Several mouse models and human syndromes caused by mutations in DNA repair and DNA damage response genes have been used to demonstrate experimentally that defects in the response to DNA damage promote progressive neurodegeneration (El-Khamisy, 2011; Sepe et al., 2016). These pathological conditions and animal disease models can be grouped into two main categories: (1) defects in processing dsDNA or ssDNA breaks or (2) defects that interrupt or inhibit completion of DNA repair pathways. Our study implicates BER as a source of DNA repair intermediates that drive normal aging. Furthermore, we show that BER activity contributes to pathology in a *C. elegans* model of PD.

With respect to neuronal aging, the present study underscores that reduced activity of BER DNA glycosylases can be neuroprotective. One of the best-characterized models demonstrating this relationship is the alkyladenine-DNA glycosylase (AAG)-deficient mouse model, which displays protection from alkylating agent-induced DNA damage (Calvo et al., 2013). Recent studies of the pathways involved in AAG-induced retinal degeneration revealed potentially translatable therapeutic approaches (Allocca et al., 2019). Similarly, it has been reported that ischemic injury is less severe (Ebrahimkhani et al., 2014), and inflammation can be reduced (Yu et al., 2016) in DNA glycosylase-deficient cells. The data presented here demonstrate improved survival of DA neurons in old PD nematodes in the absence of exposure to DNA-damaging agents. We conclude that NTH-1 generates genomic stress that drive loss of DA neurons during normal aging. Although ssDNA breaks can be generated by several cellular mechanisms, the findings of this study implicate a role for an imbalanced flux through the BER pathway as a driver of neurodegeneration. In *nth-1*;BY273 animals, which cannot initiate BER in response to oxidative DNA damage, we observed that age-dependent accumulation of ssDNA breaks was suppressed with concomitant reduction of protein PARylation. This was accompanied by improved survival of DA neurons in older NTH-1-deficient PD nematodes. This is supporting the notion that BER intermediates play a direct role in age-dependent neuronal loss in PD nematodes. Consistently, Olaparib, a well-known PARP1 inhibitor, conferred neuroprotection in PD animals, but not in *nth-1* PD animals. The observation that Olaparib has benefit and reduces neurodegeneration in models of PD suggests that the classic DNA damage response plays a role in neuronal death. Constitutive activation of PARP1 can promote neuronal damage and/or loss via NAD^+^ depletion leading to progressive mitochondrial damage, mitochondrial network fragmentation, and compensatory induction of mitophagy (Fang et al., 2016). Interestingly, although treating PD animals with NR reduced α-synuclein-induced neurotoxicity, the beneficial effect was much lower in *nth-1* mutants than in control animals. This finding suggests that NR, which is currently being tested in clinical trials of PD in humans (ClinicalTrials.gov Identifier: NCT03568968), may not be effective in all patients.

With regard to the mechanism by which NTH-1 deficiency promotes neuroprotection, we show that *nth-1*;BY273 mutants experience constitutive, mild mitochondrial stress with no change in mitochondrial network morphology, which has also been observed in nematodes with other DNA repair mutations, including NER defective *xpa-1* (Fang et al., 2014) and *csb-1* (Scheibye-Knudsen et al., 2016) and dsDNA break repair defective *atm-1* (Fang et al., 2016). However, as these mutant nematodes are deficient in NER or DSBR, they are expected to accumulate different types of DNA repair intermediates than BER-deficient animals, and primarily in the nuclei. NTH-1 is active in both mitochondria and the nucleus, and we observed fewer ssDNA breaks in nuclear DNA and higher mitochondrial DNA integrity in *nth-1* mutants. Thus, our findings demonstrate a role for NTH-1 initiated BER in both compartments as a driver of aging-related neurodegeneration. Our data also suggest that the mitochondrial phenotype associated with NTH-1 deficiency might be a direct effect of defective BER in mitochondria. In particular, we found that loss of NTH-1 activity modulated mitochondrial transcription output. In general, there is limited knowledge on mitochondrial transcription regulation (Bouda et al., 2019), and it is not known whether DNA glycosylases regulate mitochondrial transcription as has been suggested for nuclear transcription (Bordin et al., 2021). The dependency of mitohormesis on HMG-5 suggests that deregulation of transcription might be a direct cause of the mild alterations in mitochondrial metabolism in *nth-1* mutants. Optimal assembly and function of oxidative phosphorylation complexes requires coordinated mitochondrial gene expression and the availability and import of nuclear-encoded factors (Tang et al., 2020). Although these mechanisms have been mostly studied in yeast and human cells, they are likely active in *C. elegans* as well. Thus, in our model, increased mitochondrial transcripts of Oxphos proteins may generate a stress condition that promote mitohormesis without reaching a toxic level to evoke the unfolded protein response. Alternatively, transcriptional mutagenesis due to the presence of unrepaired base damage in the nth-1 mutant background might lead to accumulation of dysfunctional transcripts (Kuraoka et al., 2003). As a consequence, *nth-1* mutants exhibit a high level of hydrogen peroxide, which, in turn, stimulates mitohormesis through an LMD-3/JNK-1/SKN-1-dependent signaling cascade. JNK-1 is known to protect against oxidative stress in neurons in general (Oh et al., 2005) and in DA neurons challenged by 6-OHDA (Offenburger et al., 2018). JNK1 acts as an upstream regulator of OXR1 in *Anopheles gambiae* (Jaramillo-Gutierrez et al., 2010) and silk worms (Su et al., 2017). OXR1, the human ortholog of LMD-3, is a transcription factor activated by hydrogen peroxide that induces expression of several stress responsive genes and regulates apoptosis through p21 (Yang et al., 2014). LMD-3 might be a proximal cellular biosensor of hydrogen peroxide in the cytoplasm or mitochondria, with JNK-1 and SKN-1 activation as secondary events. However, our findings suggest that LMD-3/JNK-1/SKN-1 act as part of an orchestrated mitohormetic response, to prevent the potentially deleterious consequences of defective DNA repair and mitochondrial dysfunction.

In summary, this study demonstrates that BER is an age-dependent modifier of neuronal physiology and viability. Accumulation of mitochondrial and nuclear DNA repair intermediates due to incomplete or inefficient BER can drive neurodegeneration in PD nematodes (Figure 7). NTH-1 deficiency prevents accumulation of BER intermediates and alters mitochondrial metabolism leading to increased production of H_2_O_2_. In turn, LMD-3, JNK-1, and SKN-1 orchestrate a mitohormetic response that protects DA neurons from α-synuclein-induced neurotoxicity. The tight evolutionary conservation of the regulatory factors in this neuroprotective pathway, and the enrichment of genetic variants in human genes encoding BER components, in particular a NEIL2 variant, in human PD patients was intriguing, although this finding must be confirmed in larger cohorts and supported by further experiments. Of note, NTH1 and NEIL2 have overlapping substrate specificities, and both are bifunctional DNA glycosylases that are active in mitochondria and the nucleus. Interestingly, both NTH1 and NEIL2 have been suggested to function in transcription-associated BER and regulate nuclear gene expression (Bordin et al., 2021). Future studies are needed to define whether the modulation of mitochondrial transcription regulation is, as suggested by our data, a critical feature in diseases like PD where oxidative damage is prominent.

## STAR★Methods

### Key resources table

**Table:** 

Reagent or resource	Source	Identifier
**Antibodies**		

Mouse monoclonal 8-oxo-dG	Trevigen	Cat#4354-MC-050 RRID:AB_1857195
pJNK-1 (T183/Y185)	Novus	Cat#NBP1-72242 RRID:AB_11023348
Alexa Fluor 555-conjugated anti-rabbit	Invitrogen	Cat# A32794; RRID:AB_2762834
Alexa Fluor 555-conjugated anti-mouse	Invitrogen	Cat# A32773; RRID:AB_2762848
Anti-PAR Polyclonal	Trevigen	Cat# 4336-APC-050; RRID:AB_10643399

**Bacterial and virus strains**		

*Escherichia coli* OP50	http://www.wormbook.org/chapters/www_strainmaintain/strainmaintain.html	N/A
HT115 (DE3)	http://www.sciencedirect.com/science/article/abs/pii/S0378111900005795	N/A

**Chemicals, peptides, and recombinant proteins**		

6-OHDA	Sigma-Aldrich	Cat# H4381 CAS:28094-15-7
MPTP	Sigma-Aldrich	Cat# M0896 CAS:23007-85-4
AZD2281 PARP1 inhibitor	BPS Biosciences	Cat#27003 CAS:763113-22-0
Serotonin Hydrochloride	Sigma-Aldrich	Cat#H7752-1G CAS:61-47-2
AZD2281 (Olaparib)	BPS Bioscience	Cat#27003 CAS: 763113-22-0
Tetramethylrhodamine, ethyl ester, perchlorate (TMRE)	ThermoFisher Scientific	T669
Annexin V	Abcam	Cat# ab108194; RRID:AB_10863755

**Critical commercial assays**		

TUNEL assay kit	Invitrogen	C10619
Dopamine hydrochloride	Sigma-Aldrich	H8502
Maxima H Minus First Strand cDNA Synthesis Kit	Invitrogen	K1681
Syber Green	Applied Biosystem	4367659
EvaGreen Supermix	Bio-rad	1864034
Droplet generation oil for EvaGreen	Bio-rad	1864005

**Experimental models: Organisms/strains**		

*C. elegans:* Strain RB877: *nth-1(ok724)III*	Caenorhabditis Genetics Center	WB Strain: RB877 WormBase: WBVar00092006
*C. elegans:* Strain HLN 101: *ung-1(qa7600) III*	Nilsen Lab	
*C. elegans:* Strain TU3401: *sid-1(pk3321)V;uls69V*	Caenorhabditis Genetics Center	WB Strain: TU3401 WormBase: WBVar00239446
*C. elegans:* Strain SJ4103: N2;*Is*[p_*myo-3*_mtGFP]	Caenorhabditis Genetics Center	WB Strain: SJ4103 WBTransgene00005184
*C. elegans:* Strain SJ4103;RB877: N2;*Is*[p_*myo-3*_mtGFP]; *nth-1(ok724)III*	This Paper	-NA-
*C. elegans:* Strain NR222: kzIs9 [(pKK1260) lin-26p::NLS::GFP + (pKK1253) lin-26p::rde-1 + rol-6(su1006)].	Caenorhabditis Genetics Center	WB Strain: NR222 WormBase: WBStrain00029103
*C. elegans:* Strain: BY273; NR222	This Paper / Tavernarakis lab	
*C. elegans:* Strain: *nth-1(ok724)III*; BY273; NR222	This Paper / Tavernarakis lab	
*C. elegans:* Strain VP303: kbIs7 [nhx-2p::rde-1 + rol-6(su1006)].	Caenorhabditis Genetics Center	WB Strain: VP303 WormBase: WBStrain00040175
*C. elegans:* Strain: BY273; VP303	This Paper / Tavernarakis lab	
*C. elegans:* Strain: *nth-1(ok724)III*; BY273; VP303	This Paper / Tavernarakis lab	
*C. elegans:* Strain UA196: sid-1(pk3321); baIn11 (Pdat-1:: α-syn, Pdat-1::GFP); baIn33 (Pdat-1::sid-1, Pmyo-2::mCherry)	**Guy Caldwell Lab**	N/A
*C. elegans:* Strain: BY273	**Randy D. Blakely Lab**	N/A
*C. elegans:* Strain: BY273;RB877	This Paper/Nilsen Lab	N/A
*C. elegans:* Strain: BY273;TU3401	This Paper/Nilsen Lab	N/A
*C. elegans:* Strain: BY273;RB877;TU3401	This Paper/Nilsen Lab	N/A
*C. elegans:* Strain: IR2646: *Is*[p_*rpl-17*_Hyper]	Tavernarakis lab	
*C. elegans:* Strain: BY273; HLN 101	Nilsen Lab	
*C. elegans:* Strain: BR5270: *byIs161*[p_*rab-3*_F3(delta)K280; p_*myo-2*_mCherry]	Caenorhabditis Genetics Center	WB Strain: BR5270 WormBase: WBStrain00003901
*C. elegans:* Strain: *nth-1(ok724)III;*BR5270	This Paper / Tavernarakis lab	
*C. elegans:* Strain: IR2647: *nth-1(ok724)III;Is*[p_*rpl-17*_Hyper]	This Paper / Tavernarakis lab	
*C. elegans:* Strain: IR308: N2; *Ex001*[p_*mec-7*_GFP::LGG-1;*rol-6(su1006)*]	Tavernarakis lab	
*C. elegans:* Strain: IR2457: *nth-1(ok724)III*;*Ex001*[p_*mec-7*_GFP::LGG-1; *rol-6(su1006)*]	This Paper / Tavernarakis lab	
*C. elegans:* Strain: IR2379: N2; *Ex010*[p_*rab-3*_DsRed::LGG-1]	Tavernarakis lab	
*C. elegans:* Strain: VK2878: vkIs2878[p_*nhx-2*_CemOrange2::LGG-1; p_*myo-2*_GFP]	Caenorhabditis Genetics Center	WB Strain: VK2878 WormBase: WBStrain00040073
*C. elegans:* Strain: IR1380: N2;Ex009[p_*myo-3*_DsRed::LGG-1; p_*myo-2*_GFP]	This Paper / Tavernarakis labs	
*C. elegans:* Strain: IR2469: *nth-1(ok724)III*;*Ex010*[p_*rab-3*_DsRed::LGG-1]	This Paper / Tavernarakis lab	
*C. elegans:* Strain: IR1864: N2;*Ex001*[p_*unc-119*_TOMM-20::Rosella;*rol-6(su10006)]*	Tavernarakis lab	
*C. elegans:* Strain: IR2394: *nth-1(ok724)III*;*Ex001*[p_*unc-119*_TOMM-20::Rosella;*rol-6(su10006)*].	This Paper / Tavernarakis lab	
*C. elegans:* Strain: EG6531: N2; *oxIs608*[p_*unc-47*_mCherry]; *oxEx1182*[p_*unc-47*_TOMM-20::GFP]	Eric Jorgensen Laboratory	
*C. elegans:* Strain: IR2395: *nth-1(ok724); oxIs608*[p_*unc-47*_mCherry]; *oxEx1182*[p_*unc-47*_TOMM-20::GFP]	This Paper / Tavernarakis lab	
*C. elegans:* Strain: IR2648: *unc-119(ed3); Is013*[p_*myo-3*_NTH-1::GFP; *unc-119(+)*]	This Paper / Tavernarakis lab	
*C. elegans:* Strain: HLN111: N2; *repEx1*(p_*myo-3*_NTH-1(117bp)::GFP)	This Paper/Nilsen Lab	N/A
*C. elegans:* Strain: HLN114: N2; *repEx4*(p_*myo-3*_NTH-1(1st Exon)::GFP)	This Paper/Nilsen Lab	N/A

**Oligonucleotides**		

FW *nth-1*: GGATCCATGCATTCTTTCAGTATG AAACGAGTT	This Paper	
RV *nth-1*: ACCGGTTCCGTTTCCGATTCAGTT TTCACTTC	This Paper	
FW atp-6: TTGTCCTTGTGGAATGGTTGA	This Paper	
RV *atp-6*: TTTCAAATATGTGTCTCCTGGTTTTC	This Paper	
FW *ndlf-4*: TGACAACGTTTAATTTTTATTCTA ATTTCTTTAG	This Paper	
RV *ndlf-4*: ACTACCATACCCAGGATTCTTGAAA	This Paper	
FW *ctc-1*: TTGGGATTTTCACGGGTGTT	This Paper	
RV *ctc-1*: TGCAAAATGTAGCGGGAAAA	This Paper	
FW *nduo-2*: AAAGCAGCAAGAGATATACCAGAATTT	This Paper	
RV *nduo-2*: TCAAAAGTGGAGCGGTGCTA	This Paper	
FW *pmp-3*: GGAACTTAGAGTCAAGGGTCGCAG	This Paper	
RV *pmp-3*: GAACTGTATCGGCACCAAGGAAACTG	This Paper	
FW ND1: AGCGTCATTTATTGGGAAG AAGAC	This Paper	
RV ND1: AAGCTTGTGCTAATCCCATAAATGT	This Paper	
FW *nth-1* N terminus: catgGGATCCATGCATTCT TTCAGTATGAAACGAGTTGTT	This Paper	
Rv *nth-1*-mito (117bp): catgGGTACCTTTCGAAT TAATTCCACGTCACGTCT	This Paper	
Rv *nth-1*-mito (1^st^ Exon): catgGGTACCGGCGG AGCTGCCAAAGG	This Paper	

**Recombinant DNA**		

pPD96.52	Addgene	Fire lab C. elegans vector kit
pJA327	Addgene	Addgene plasmid # 74486
NTH-1::GFP in pPD96.52	This Paper / Tavernarakis lab	
P_myo-3_NTH-1(117bp)::GFP	This Paper / Nilsen lab	
P_myo-3_NTH-1(1^st^ Exon)::GFP	This Paper / Nilsen lab	
*skn-1* in pL4440	Tavernarakis lab	
*jnk-1* in pL4440	Tavernarakis lab	
*hmg-5* in pL4440	Tavernarakis lab	
*nth-1* in pL4440	This Paper / Tavernarakis lab	
*sod-1* in pL4440	This Paper / Tavernarakis lab	
*sod-2* in pL4440	This Paper / Tavernarakis lab	
*sod-3* in pL4440	This Paper / Tavernarakis lab	

**Software and algorithms**		

Biorender	Cell Press	
GraphPad Software Inc., San Diego, USA	GraphPad Software	
Zen	Zeiss Software	
Fiji	Fiji software	

**Other**		

Axio-Imager Z2 epifluorescence microscope	Zeiss	
Axio-Observer Z1/ LSM710 NLO/ DUO/InTune multiphoton confocal microscope	Zeiss	
EVOS FL Auto 2 Cell imaging systems	ThermoFisher Scientific	

### Resource availability

#### Lead contact

Further information and request for resources and reagents should be directed to and will be fulfilled by the Lead Contact, Hilde Nilsen (h.l.nilsen@medisin.uio.no).

#### Material availability

All unique/stable reagents generated in this study are available from the Lead Contact, Hilde Nilsen (h.l.nilsen@medisin.uio.no) without restriction.

### Experimental model and subject details

#### *C. elegans* strains and culture conditions

Animals were grown on nematode growth medium (NGM) plates with *Escherichia coli* strain OP50 at 20°C using standard procedure (Brenner, 1974). To obtain synchronize population adult worms were bleached. For all aging associated studies L4 hermaphrodites were grown in NGM plates without FUDR, old worms were maintained by filtering using nylon filter. The adult Day 1 was defined as 24 hours post L4 stage.

### Method details

#### Molecular cloning

To generate the p_*myo-3*_NTH-1::GFP reporter construct, we fused a BamHI*/AgeI* fragment, containing the coding sequence of *nth-1* without the stop codon, amplified from *C. elegans* genomic DNA using the primers 5′-GGATCCATGCATTCTTTCAGTATGAAACGAGTT-3′ and 5′- ACCGGTTCCGTTTCCGATTCAGTTTTCACTTC-3′, at the amino (N) terminus of GFP, in the pPD96.52 plasmid vector. The p_*myo-3*_NTH-1::GFP fusion construct was co-bombarded with the rescue plasmid of *unc-119(+)* in HT1593 strain. To generate the constructs P_myo-3_NTH-1(117bp)::GFP and P_myo-3_NTH-1(1^st^ exon)::GFP, we fused BamHI*/KpnI* fragments containing the first 117bp of *nth-1* coding sequence and its first exon, amplified from *C. elegans* genomic DNA using the primers 5′- catgGGATCCATGCATTCTTTCAGTATGAAACGAGTTGTT −3′, 5′- catgGGTACCTTTCGAATTAATTCCACGTCACGTCT −3′ and 5′- catgGGTACCGGCGGAGCTGCCAAAGG −3′ at the amino (N) terminus of GFP, in the pJA327 plasmid vector. pJA327 was a gift from Andrew Fire (Addgene plasmid # 74486 ; http://addgene.org/74486; RRID:Addgene_74486). The P_myo-3_NTH-1(117bp)::GFP and P_myo-3_NTH-1(1^st^ exon)::GFP constructs were injected in the N2 strain at 30ng/μL. For engineering the *skn-1, nth-1, sod-1, sod-2* and *sod-3* RNAi constructs, gene-specific fragments of interest were obtained by PCR amplification directly from *C. elegans* genomic DNA using gene-specific sets of primers. The PCR-generated fragments were sub-cloned into the pL4440 plasmid vector. The resulting constructs were transformed into HT115(DE3) *Escherichia coli* bacteria deficient for RNase III. Bacteria carrying an empty vector were used in control experiments.

#### Degeneration of dopaminergic neurons

The degeneration of dopamine neurons in BY273 *Is*[*p*_*dat-1*_GFP; p_*dat-1*_ α-syn] and BY273;*nth-1 Is*[*p*_*dat-1*_GFP; p_*dat-1*_ α-syn];*nth-1 (ok724)III* was monitored by following dopaminergic neurons specific GFP expression under dopamine transporter (dat-1) promoter. For neurotoxicity assay BY273 and BY273;*nth-1* worms at L4 larval stage was exposed to 30mM 6-OHDA and 2mM MPTP for 48 hours. The aging population of BY273 and BY273;*nth-1* worms were maintained in NGM plates without FdUrd, instead we filtered worms every day after day 2 adult stage with Nylon Net Filter (catalog #NY4104700) till the desired age we wanted. Here, we have mostly harvested the worms on day 5 or day 7 old adult stage. At this stage 15-20 worms were immobilized in agar padded glass slide with 2mM levamisole and glass coverslip. The dopaminergic (DA) neurons were imaged under Zeiss LSM780 confocal microscope with x63 plan-Apochromat 1.4 NA objective. The RNAi treated young and old worms were imaged under x 10 objective with 2.4x zoom.

#### Mitochondrial imaging

TMRE staining: Tetramethylrhodamine, ethyl ester, perchlorate is a dye that accumulates in intact, functional mitochondria. 1-day-adult animals were grown at 20°C in the presence of 150 nM TMRE for 24 h. Stained and washed worms were immobilized with levamisole before mounting on 2% agarose pads for microscopic examination with a Zeiss AxioImager Z2 epifluorescence microscope. Images were acquired under the same exposure. Average pixel intensity values were calculated by sampling images of different animals. We calculated the mean and maximum pixel intensity for each animal using the Fiji software (https://fiji.sc). For each experiment, at least 35 animals were examined for each strain/condition. Each assay was repeated at least three times. We used the Prism software package (GraphPad Software) for statistical analyses.

#### Dopamine resistance assay

For DA resistance assay, 1-day-old adult hermaphrodites were incubated in a droplet of 20 μL of M9 buffer containing DA hydrochloride (Sigma-Aldrich) at a final concentration of 40 mM. Animal were scored for paralysis every 10 minutes. Nematodes were considered paralyzed when they did not exhibit any spontaneous body bend during a period of 5 s. Three distinct populations of 20 adults (for each strain) were scored over the assay period. We performed three independent measurements per strain. We used the Prism software package (GraphPad Software) for statistical analysis.

#### TUNEL assay

The TUNEL staining was performed using Click-iT® Plus TUNEL Assay kit (Invitrogen). The worms were collected from day 1 young adults and day 7 old adults and then washed with sterile milliQ water before fixing with 4% PFA. The fixed worms were further permeabilized using proteinase K from the kit, followed by TdT and click-iT reaction as mentioned in the user guide. Alexa Fluor® 647 secondary antibody was used from the kit. For mounting prolong gold with DAPI was used to detect the DNA (Invitrogen, P36931). The slides were imaged under Zeiss LSM780 confocal microscope with x63 plan-Apochromat 1.4 NA objective.

#### Immunohistochemistry

For immunostaining, day 1 young and day 7 old adult worms were washed with milliQ water twice. Washed worms were placed on poly lysin-coated slides (Thermo Scientific), and freeze cracked using coverslip on dry ice block. The immunostaining was performed as described by us previously (Kassahun et al., 2018; SenGupta et al., 2013). Briefly, animals on the slides were fixed in acetone and methanol in 1:1 ratio for 10 min at −20°C, washed in PBS-T (1 x PBS, 0.1% Tween-20) for 5 min, followed by 30 min blocking in PBS-TB (1 x PBS, 0.1% Tween-20, 0.5% BSA). The slides were incubated with primary antibody overnight at 4°C, next day washed three times for 10 min in PBS-T, followed by incubation with the secondary antibody at room temperature for 2 h. Primary antibody used at following dilutions: pJNK-1(1:200), 8-oxo-dG (1:200), PAR (1:200) and Annexin V (1:200). The secondary antibody used to detect was Alexa Fluor 555-conjugated anti-rabbit and anti-mouse (1:1500). For mounting prolong gold with DAPI was used to detect the DNA (Invitrogen, P36931). The slides were imaged under Zeiss LSM780 confocal microscope with x63 plan-Apochromat 1.4 NA objective).

#### HPLC-MS/MS quantification of DNA

An antioxidant - butylated hydroxytoluene (100 μM) and internal standards: ^13^C^15^N-5-oh(dC) (1nM) and ^15^N_3_^13^C_2_-8-oxo(dG) (5 nM) were added to the samples before processing. DNA was digested to nucleosides by nuclease P1 from *Penicillium citrinum* (Sigma, N8630), benzonase (Santa Cruz Biotech, sc-391121B) and AP from *E. coli* (Sigma P5931) in 10 mM ammonium acetate buffer pH 6.0, 1 mM MgCl_2_ for 40 min at 40°C. Three volumes of acetonitrile were added to hydrolysates and samples were centrifuged (16,000 g, 30 min, 4°C). The supernatants were lyophilized and dissolved in 50 μl water for HPLC-MS/MS analysis. Chromatographic separation was performed using an Agilent 1290 Infinity II UHPLC system with an ZORBAX RRHD Eclipse Plus C18 150 × 2.1 mm ID (1.8 μm) column protected with an ZORBAX RRHD Eclipse Plus C18 5 × 2.1 mm ID (1.8 μm) guard column (Agilent). The mobile phase consisted of A: water and B: methanol (both added 0.06% acetic acid or 0.1% formic acid for separation of 5-hm(dU) or all the other nucleosides, respectively). In case of 5-hm(dU) chromatography run started at 0.25 ml/min flow of 5% B for 0.5 min and was followed by 3.5 min gradient of 5%–20% B, 1 min of 20%–80% B, 2 min of washing with 80% B, 0.5 min gradient of 80%–5% B at 0.27 ml/min ending with 4 min re-equilibration with 5% B. For other modifications run started at 0.15 ml/min flow of 5% B for 3 min followed by 0.5 min gradient of 5%–13% B at 0.15 ml/min, 3 min of 13%–17% B at 0.2 ml/min, 1.5 min of 55%–80% B at 0.25 ml/min, 1.5 min of 80%–50% B at 0.25 ml/min and 1 min of 50%–5% B at 0.25 ml/min, ending with 4 min re-equilibration with 5% B at 0.25 ml/min. Unmodified nucleosides were chromatographed isocratically with 20% B at 0.23 ml/min. An Agilent 6495 Triple Quadrupole system operating in positive electrospray ionization mode was used for mass spectrometric detection. The following mass transitions were monitored: 258.1/142.1 (5-hm(dC)); 261.1/145.1 (D_3_-5-hm(dC)); 284.1/168.1 (8-oxo(dG)); 289.1/173.1 (^15^N_3_^13^C_2_-8-oxo(dG)); 244.1/128.1 (5-oh(dC)); 247.1/131.1 (^13^C^15^N-5-oh(dC)); 259.1/142.1 (5-hm(dU)); 252.1/136.1 (dA); 228.1/112.1 (dC); 268.1/152.1 (dG); 243.1/127.1 (dT).

#### Mitochondrial DNA copy number

The mitochondrial DNA (mtDNA) copy number was quantified using droplet digital PCR (ddPCR). The age synchronized N2, *nth-1*, BY273 and BY273;*nth-1* animals were individually picked in lysis buffer (TE low containing 1mg/ml proteinase K and 0,01 mg/ml RNase). The lysis was performed for 1hr in 65°C followed by 95°C for 15 minutes. Samples were immediately stored at −80°C. Before running the ddPCR, 29μl of nuclease free water was added after two cycles of freeze thaw for 5 minutes each. The lysates were further diluted (1:100) and assayed using Droplet Digital PCR QX system (Bio-Rad). Briefly, the diluted lysate was added to a PCR mixture containing 2x QX200 ddPCR EvaGreen Supermix (Bio-Rad) and 100 nM ND1 primers. Then, the 20 μL of PCR mixture and 70 μL Droplet generation oil for EvaGreen (Bio-Rad) were mixed. Droplets were generated using a QX100 Droplet Generator (Bio-Rad). The following PCR conditions were used after denaturing at 95°C for 5 min, 40 cycles at 95°C for 30 s and 60°C for 1 min were followed by 1 step at 4°C for 5 min and 90°C for 5 min. The cycled droplets were read in the QX200 Droplet Reader (Bio-Rad), and analyzed with QuantaSoft droplet reader software.

#### Mitochondrial gene expression analysis

Transcriptional activation of *atp-6*, *ndfl-4*, *ctc-1* and *nduo-2* was measured in age synchronized N2, *nth-1*, BY273 and BY273;*nth-1* animals. Single worms were collected in 1 μl PBS and immediately snap frozen in dry ice. cDNA synthesis was performed using Maxima H Minus First Strand cDNA Synthesis Kit, with dsDNase, according to the manufacturer’s instructions. Quantitative reverse transcriptase PCR (qRT-PCR) was performed with SYBR Green supermix (Bio-Rad). *atp-6*, *ndfl-4*, *ctc-1* and *nduo-2* transcript levels were normalized to *pmp-3* housekeeping gene. Mitochondrial gene expression was quantified by further normalizing the fold change with mitochondrial copy number for each gene.

#### Basal slowing response

The basal slowin response was performed as described by (Sawin et al., 2000) with minor modification suggested by (Wang et al., 2018a). Only well-fed synchronized young day 1 and old day 5 adult worms maintained at 20°C were tested. The NGM plates with and without OP50 were used to count body bends per 20 s. We started counting the locomotion rate after 5 minutes of transfer, to avoid overstimulation. This assay was performed blindfolded, with three independent replicates.

#### Pharyngeal pumping

We used a recently developed micro fluids based ScreenChip system from NemaMatrix to detect pharyngeal pumping rate in each individual worm. For this assay worms were age synchronized, day 1 young and day 7 old worms were used. The worms were first washed twice with M9 buffer, before incubating them with 10 mM serotonin at room temperature for 20 minutes. Simultaneously, the NemaMatrix fluidics system was prepared by following the user guide then worms were loaded in Eppendorf, through fine tubing’s and vacuum pump worms were sucked to the ScreenChip. From the rightly positioned worm the pumping frequency was measured from EPG recording using NemaMatrix ScreenChip 40 and 60 for young and old worms respectively (Harris et al., 2019). Each recording was 1 to 2 minutes long, with 15-20 worms per genotype.

### Progeny assay

This assay was performed as described in (Wang et al., 2018a). For each genotype we had five L4 larva, each larvae were transferred to single NGM plate with OP50 food in the center. After 24 hours, number of eggs were counted and adult were transferred to fresh plates, until the adults stopped laying any further eggs. The live progeny’s were also counted subsequently in each well.

#### Genetic association analyses

Whole-exome sequencing was performed on all individuals with clinically validated PD (n = 192) from the Norwegian ParkWest study, a prospective population-based cohort of idiopathic PD (PMID: 19246476). Controls (n = 219) were provided from cohorts of previously sequenced individuals with testis cancer (n = 167) or acoustic neuroma (n = 52) who had been recruited and examined at Haukeland University Hospital and had no clinical signs of neurodegenerative or other neurological disorders. DNA was extracted from blood by routine procedures and sequenced at HudsonAlpha Institute for Biotechnology (Huntsville, AL), using Roche-NimbleGen Sequence Capture EZ Exome v2 (173 controls) and v3 (all PD and 46 controls) kits and paired-end 100 bp sequencing on the Illumina HiSeq platform. The reads were mapped to the hg19 reference genome using BWA v0.6.2(PMID: 19451168), PCR duplicates removed with Picard v1.118(cite: http://broadinstitute.github.io/picard), and the alignment refined using Genome Analysis Toolkit (GATK) v3.3.0 (PMID: 20644199) applying base quality score recalibration and realignment around indels recommended in the GATK Best Practices workflow (PMID: 21478889, 25431634). Variants were called in all samples using GATK HaplotypeCaller (PMID: 20644199) with default parameters. Next, Variant Quality Score Recalibration (VQSR) was performed using 99.9% sensitivity threshold (PMID: 20644199). The remaining variants were filtered against the intersection of capture targets (v2 and v3) using BEDtools (PMID: 25199790) and VCFtools (PMID: 21653522). Variants with total depth below 10X were marked as unknown genotype (no-call) using BCFtools (PMIS: 21903627). Indel calls, which were found to be less reliable than single-nucleotide variants, were excluded from downstream analyses. WES data were recoded into binary PLINK input format, and QC of individual and SNP data was performed using PLINK v1.9026 (PMID: 25722852). Individuals were excluded if their genotypic data showed a missing rate > 2%, abnormal heterozygosity (+/− 3 standard deviations [SD], calculated for common and rare variants separately), conflicting sex assignment, cryptic relatedness (IBD > 0.2), or divergent ancestry (non-European). Population stratification was studied using multi-dimensional scaling (MDS) against the HapMap-populations (cite: http://www.nature.com/articles/nature02168). SNPs were excluded due to genotyping rate less than 98%, different call rates between cases and controls (p < 0.02) or departure from Hardy-Weinberg equilibrium (p < 0.00005). Only autosomes were considered. Monomorphic and multi-allelic variants were removed. A principal component analysis was performed using Eigensoft (PMID: 16862161, PMID: 17194218) with standard settings. An analysis of variance of the first 10 principal components was performed, and statistically significant principal components (p < 0.01) were used as covariates in all further analyses to correct for population stratification. QQ- and MDS-plots for the ParkWest dataset have been published previously (PMID: 30256453). Variants were annotated using ANNOVAR (PMID: 20601685), and nonsynonymous, stopgain, stoploss and splice variants were extracted. Only rare variants were used in the statistical analyses, and were defined as variants with a minor allele frequency < 1% in the non-Finnish European subset of gnomAD (cite doi: http://biorxiv.org/lookup/doi/10.1101/531210]). Genetic association analyses in the form of the SKAT-O test were performed using R version 3.6.1 (www.r-project.org) and the SKAT R package version 1.3.2.1 (PMID: 22863193) with default settings. P values were adjusted for multiple comparisons using the Bonferroni method.

### Quantification and statistical analysis

#### Data analysis

Zen blue software from Zeiss was used to quantify the intensity of GFP in dopaminergic neurons, pJNK-1 intensity and TUNEL positive intensity as described previously in (Kassahun et al., 2018). The pharyngeal pumping frequency was quantified using Nemametrix analysis software.

#### Statistical analysis

The statistical analysis was performed in GraphPad prism 8 software by using one-way ANOVA followed by Bonferroni’s or Šidák’s multiple comparison test (Figures 1C–1E, 2A–2C, 3C–3E, 4B–4D, 5B, 5D–5H, 6A–6D, and 6F); two-way ANOVA (3F); unpaired t test (Figures 3B, 4A, 5A, and 5C), represented as column scatterplots which indicates s.e.m from three biological replicates, where p < 0.05 is considered significant.

## Data Availability

Original data from human PD cohorts are available upon request from Charalampos Tzoulis (charalampos.tzoulis@nevro.uib.no) as detailed in the original publication (Gaare et al., 2018) No new code is reported in this paper. Any additional information required to reanalyze the data reported in this paper is available from the lead contact upon request.

## Supplemental information

Document S1. Figures S1–S8 and Tables S1 and S2

Document S2. Article plus supplemental information
