# Supplementary material for: Base excision repair causes age-dependent accumulation of single-stranded DNA breaks that contribute to Parkinson disease pathology
Source: Cell Rep. 2021 Sep 7;36(10):109668. doi: 10.1016/j.celrep.2021.109668 (PMC8441048; doi:10.1016/j.celrep.2021.109668)
Supplement: Document S1. Figures S1–S8 and Tables S1 and S2 [file mmc1.pdf]

Cell Reports, Volume *36*

Supplemental information

Base excision repair causes age-dependent accumulation of single-stranded DNA breaks that contribute to Parkinson disease pathology

Tanima SenGupta, Konstantinos Palikaras, Ying Q. Esbensen, Georgios Konstantinidis, Francisco Jose Naranjo Galindo, Kavya Achanta, Henok Kassahun, Ioanna Stavgiannoudaki, Vilhelm A. Bohr, Mansour Akbari, Johannes Gaare, Charalampos Tzoulis, Nektarios Tavernarakis, and Hilde Nilsen

Cell Reports

Base excision repair causes age-dependent accumulation of single-stranded DNA breaks that contribute to Parkinson's disease pathology

Tanima SenGupta, Konstantinos Palikaras, Ying Q Esbensen, Georgios Konstantinidis, Francisco Jose Naranjo Galindo, Kavya Achanta, Henok Kassahun, Ioanna Stavgiannoudaki, Vilhelm A. Bohr, Mansour Akbari, Johannes Gaare, Charalampos Tzoulis, Nektarios Tavernarakis and Hilde Nilsen

Supplemental information

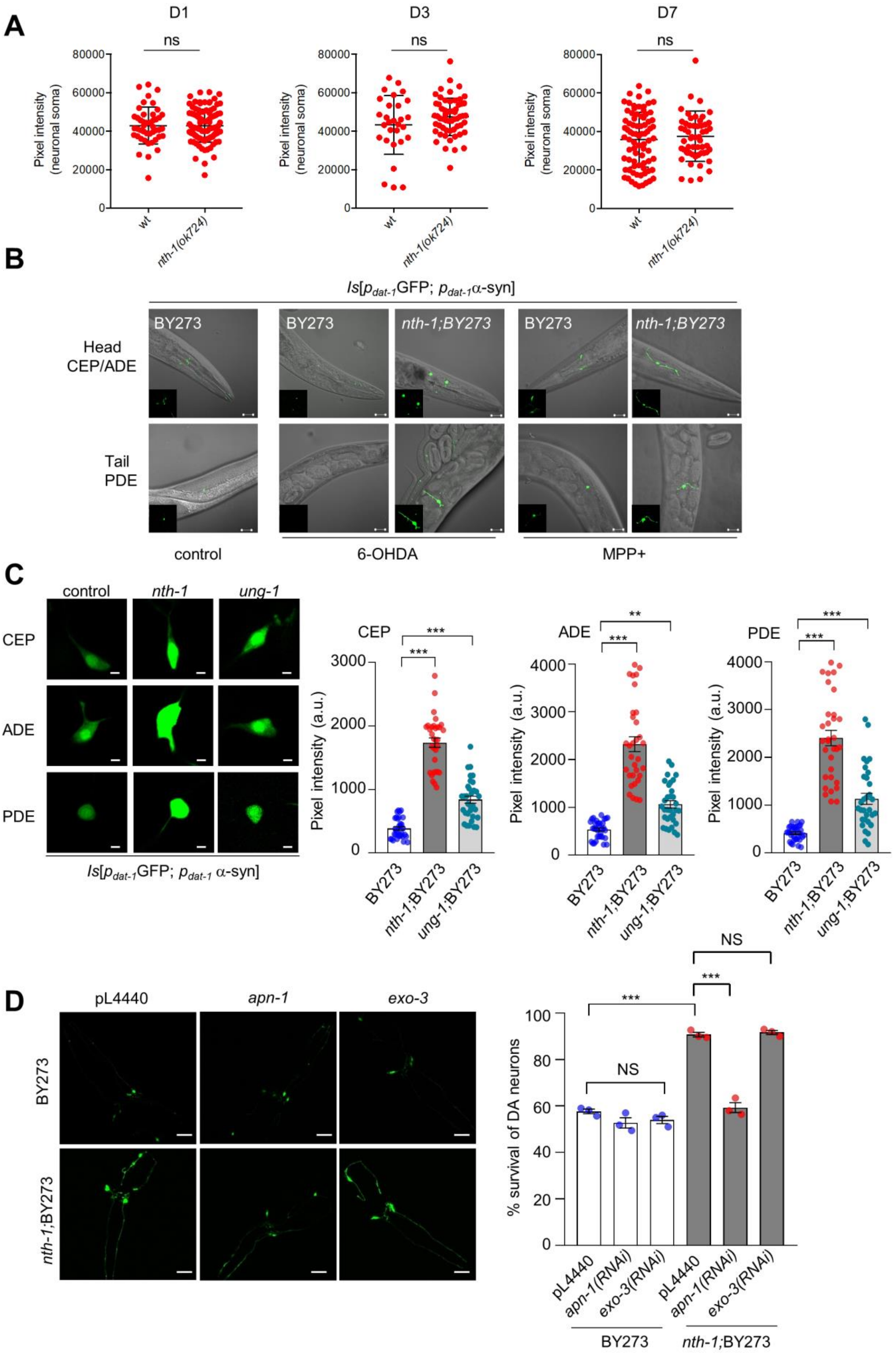
A
D1
D3
D7
ns
Pixel intensity (neuronal soma)
80000
60000
40000
20000
0
wt
nth-1(ok724)
B
Is[pdat-1GFP; pdat-1α-syn]
BY273
nth-1;BY273
Head CEP/ADE
Tail PDE
control
6-OHDA
MPP+
C
control
nth-1
ung-1
CEP
ADE
PDE
Is[pdat-1GFP; pdat-1 α-syn]
Pixel intensity (a.u.)
***
**
3000
2000
1000
4000
BY273
nth-1;BY273
ung-1;BY273
D
pL4440
apn-1
exo-3
BY273
nth-1;BY273
NS
***
% survival of DA neurons
100
80
60
40
20
0
pL4440
apn-1(RNAi)
exo-3(RNAi)

Supplementary Figure 1. NTH-1 deficiency promotes neuronal survival following exogenous stress. Related to Figure 1. (A) NTH-1 deficiency does not affect the transcriptional levels from the *dat-1* promoter during ageing (n= 40 nematodes per group; NS p>0.05; unpaired t-test). **(B)** NTH-1 deficiency protects dopaminergic neurons in response to 30mM 6-OHDA and 2mM MPP+(n= 30 from three independent repeats). **(C)** Age-dependent neurodegeneration is proficiently diminished in *nth-1;*BY273 as compared to *ung-1*;BY273 mutants (n=30 from three independent experiments; ***p<0.001; one-way ANOVA followed by Bonferroni's multiple comparison test). **(D)** Depletion of *apn-1* by RNAi abolishes age dependent neuroprotection in *nth-1;*BY273 animals, however *exo-3* depletion has no effect in *nth-1;*BY273 nematodes (n= 40 to 55 nematodes per group; **p<0.01 and ***p<0.001; one-way ANOVA followed by Bonferroni's multiple comparison test,error bars, s.e.m.).

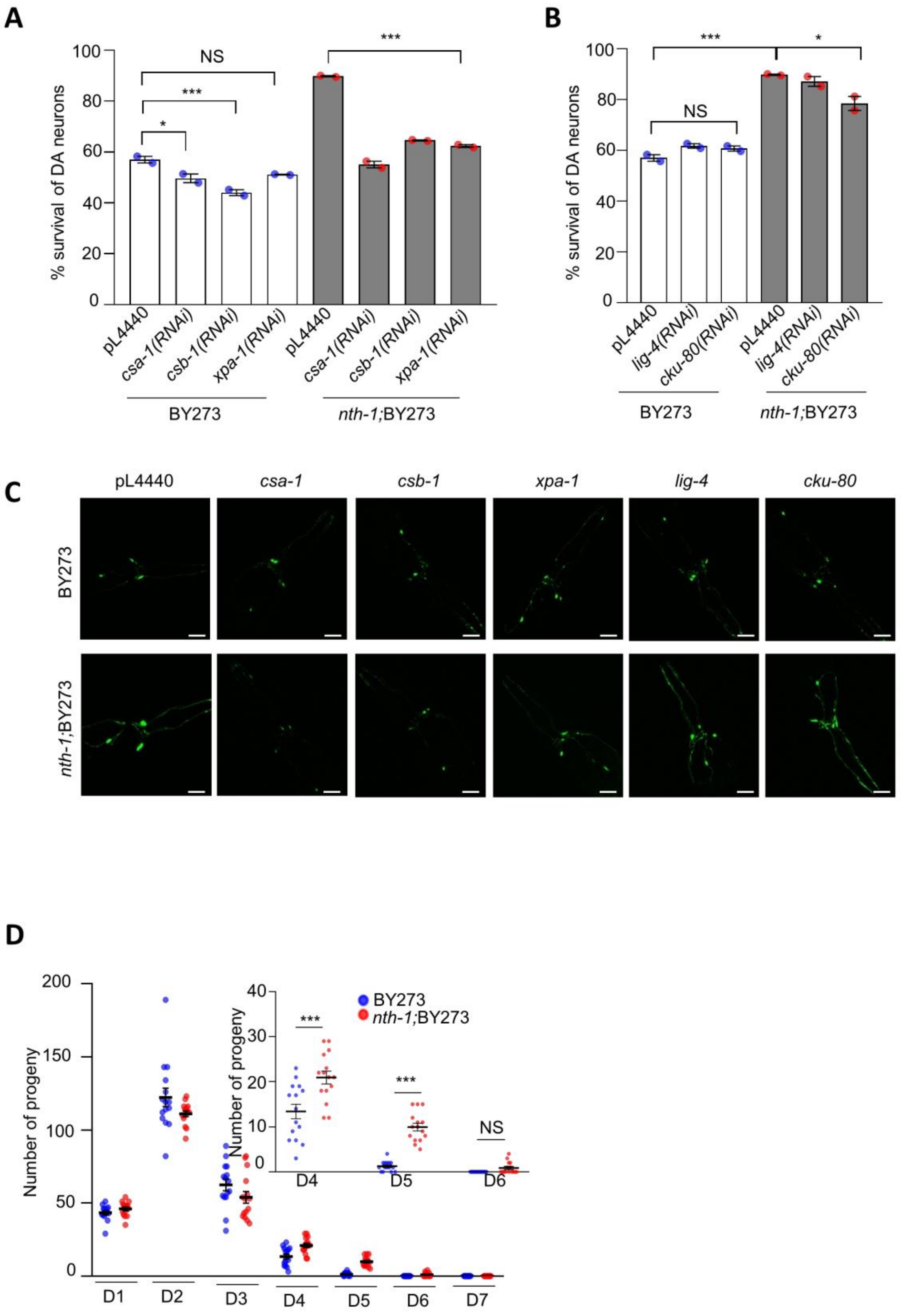
A
B
C
D
% survival of DA neurons
NS
***
*
pL4440
csa-1(RNAi)
csb-1(RNAi)
xpa-1(RNAi)
lig-4(RNAi)
cku-80(RNAi)
BY273
nth-1;BY273
pL4440
csa-1
csb-1
xpa-1
lig-4
cku-80
Number of progeny
D1
D2
D3
D4
D5
D6
D7

Supplementary Figure 2. The effect of NER and NHEJ impairment on DA neurons viability of PD nematode model. Related to Figure 1. (A) (C) Age dependent neuroprotection in *nth-1;*BY273 animals is abrogated upon knocking down of NER components like *csa-1*, *csb-1* and *xpa-1* (n= 40 to 55 nematodes per group; **p< 0.01, ***p<0.001; one-way ANOVA followed by Bonferroni's multiple comparison test). **(B) (C)** Age dependent neuroprotection in *nth-1;*BY273 animals is abrogated upon depletion of *cku-80* and shows no effect upon *lig-4* depletion(n= 30 from 2 independent experiments). Scale bar, 20μm. **(D)** The reproductive lifespan of transgenic BY273 and *nth-1;*BY273 animals. The bar graphs represent the number of eggs laid from day 1 to day 7 post L4 (n= 15; 5 individual per strain, three replicates, NS p>0.05, and ***p<0.001; one-way ANOVA followed by Bonferroni's multiple comparison test).

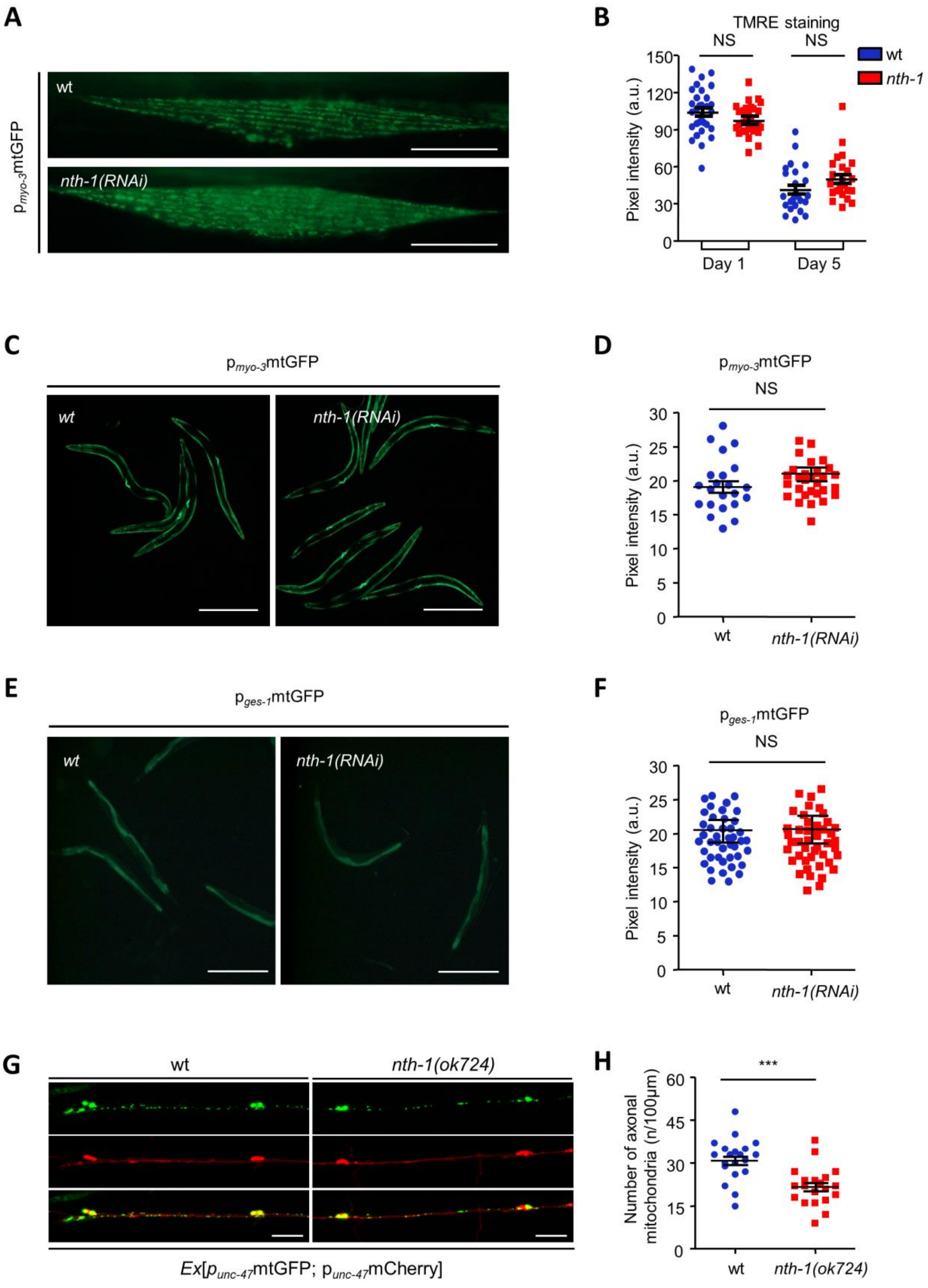

A
B
TMRE staining
NS
NS
wt
nth-1
Pixel intensity (a.u.)
Day 1
Day 5
wt
nth-1(RNAi)
pmyo-3mtGFP
C
D
pmyo-3mtGFP
wt
nth-1(RNAi)
pmyo-3mtGFP
NS
Pixel intensity (a.u.)
wt
nth-1(RNAi)
E
F
pges-1mtGFP
wt
nth-1(RNAi)
pges-1mtGFP
NS
Pixel intensity (a.u.)
wt
nth-1(RNAi)
G
H
wt
nth-1(ok724)
Ex[punc-47mtGFP; punc-47mCherry]
***
Number of axonal mitochondria (n/100μm)
wt
nth-1(ok724)

Supplementary Figure 3. Mitochondrial homeostasis is not altered in NTH-1 deficient animals. Related to Figure 2. (A) Mitochondrial network morphology is not affected upon knocking down of *nth-1.* Scale bar, 20μm. **(B)** NTH-1 deficient animals do not display mitochondrial membrane potential defects (n= 45 nematodes per group; NS p>0.05; unpaired t-test). Knocking down of *nth-1* does not affect mitochondrial mass in either body-wall muscle **(C, D)** or intestinal cells **(E, F)** (n= 25 – 40 nematodes per group; NS p>0.05; unpaired t-test). Scale bar, 500μm. **(G, H)** NTH-1 deficient animals display less axonal mitochondria in GABAergic motor neurons. Representative confocal images of mtGFP (green) and cytoplasmic mCherry (red) in GABAergic motor neurons of WT and *nth-1* transgenic nematodes (n=30 nematodes per group; ***p< 0.001; unpaired t-test). Error bars, s.e.m.

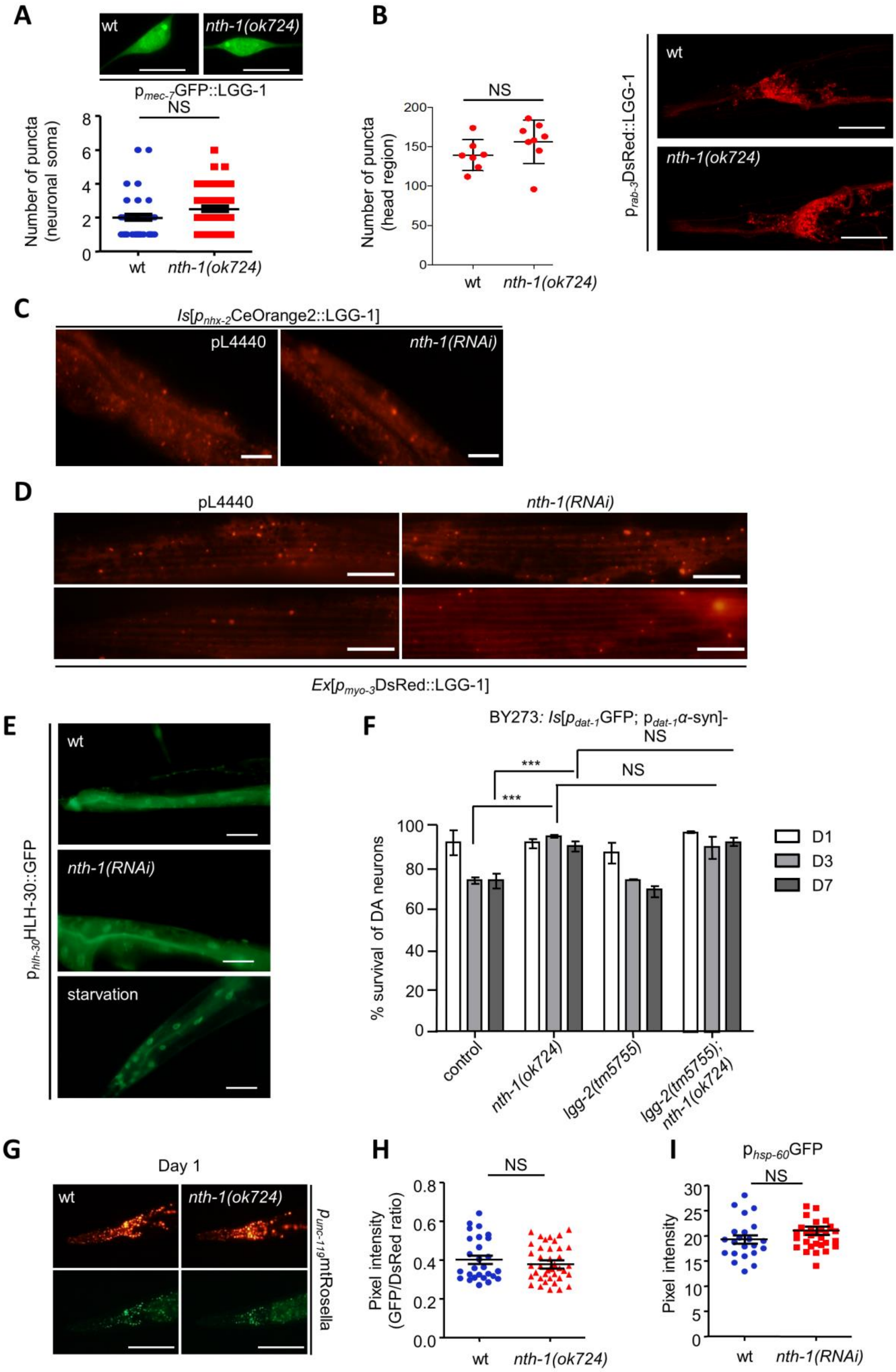
A
wt
nth-1(ok724)
pmec-7GFP::LGG-1
NS
Number of puncta (neuronal soma)
8
6
4
2
0
wt
nth-1(ok724)
B
NS
Number of puncta (head region)
200
150
100
50
0
wt
nth-1(ok724)
prab-3DsRed::LGG-1
wt
nth-1(ok724)
C
Is[pnhx-2CeOrange2::LGG-1]
pL4440
nth-1(RNAi)
D
pL4440
nth-1(RNAi)
Ex[pmyo-3DsRed::LGG-1]
E
phlh-30HLH-30::GFP
wt
nth-1(RNAi)
starvation
F
BY273: Is[pdat-1GFP; pdat-1α-syn]-
NS
***
NS
***
% survival of DA neurons
100
80
60
40
20
0
D1
D3
D7
control
nth-1(ok724)
lgg-2(tm5755)
lgg-2(tm5755); nth-1(ok724)
G
Day 1
wt
nth-1(ok724)
punc-119mtRosella
H
NS
Pixel intensity (GFP/DsRed ratio)
0.8
0.6
0.4
0.2
0.0
wt
nth-1(ok724)
I
phsp-60GFP
NS
Pixel intensity
30
25
20
15
10
5
0
wt
nth-1(RNAi)

Supplementary Figure 4. Autophagy and mitophagy are not induced in neuronal cells of *nth-1* mutants. Related to Figure 2. Transgenic animals expressing autophagosomal protein LGG-1 fused with GFP in six touch receptor neurons **(A)** or LGG-1 fused with DsRed under the pan-neuronal promoter of *rab-3* gene **(B)** or LGG-1 fused with CeOrange2 in the intestine **(C)** or LGG-1 fused with DsRed under the body-wall muscle promoter of *myo-3* gene **(D)** do not display altered autophagy levels upon NTH-1 deficiency (n= 15 – 30 nematodes per group; NS p>0.05; unpaired t-test)**. (E)** HLH-30/TFEB subcellular localization does not change upon NTH-1 depletion. **(F)** LGG-2 deficiency does not abolish neuroprotection against α-synuclein in *nth-1* mutants (n= 3 biological replicates, 40 nematodes per group; NS p>0.05, ***p<0.001; one-way ANOVA followed by Sidak's multiple comparisons test). **(G, H)** Neuronal mitophagy is not induced in response to NTH-1 deficiency. WT and *nth-1* transgenic nematodes expressing pan-neuronally mtRosella biosensor (n= 40 nematodes per group; NS p>0.05; unpaired t-test). Scale bar, 50μm. **(I)** Mitochondrial unfolded protein response (mtUPR) is not activated upon RNAi against *nth-1* (n= 20-25 nematodes per group; NS p>0.05; unpaired t-test). Error bars, s.e.m.

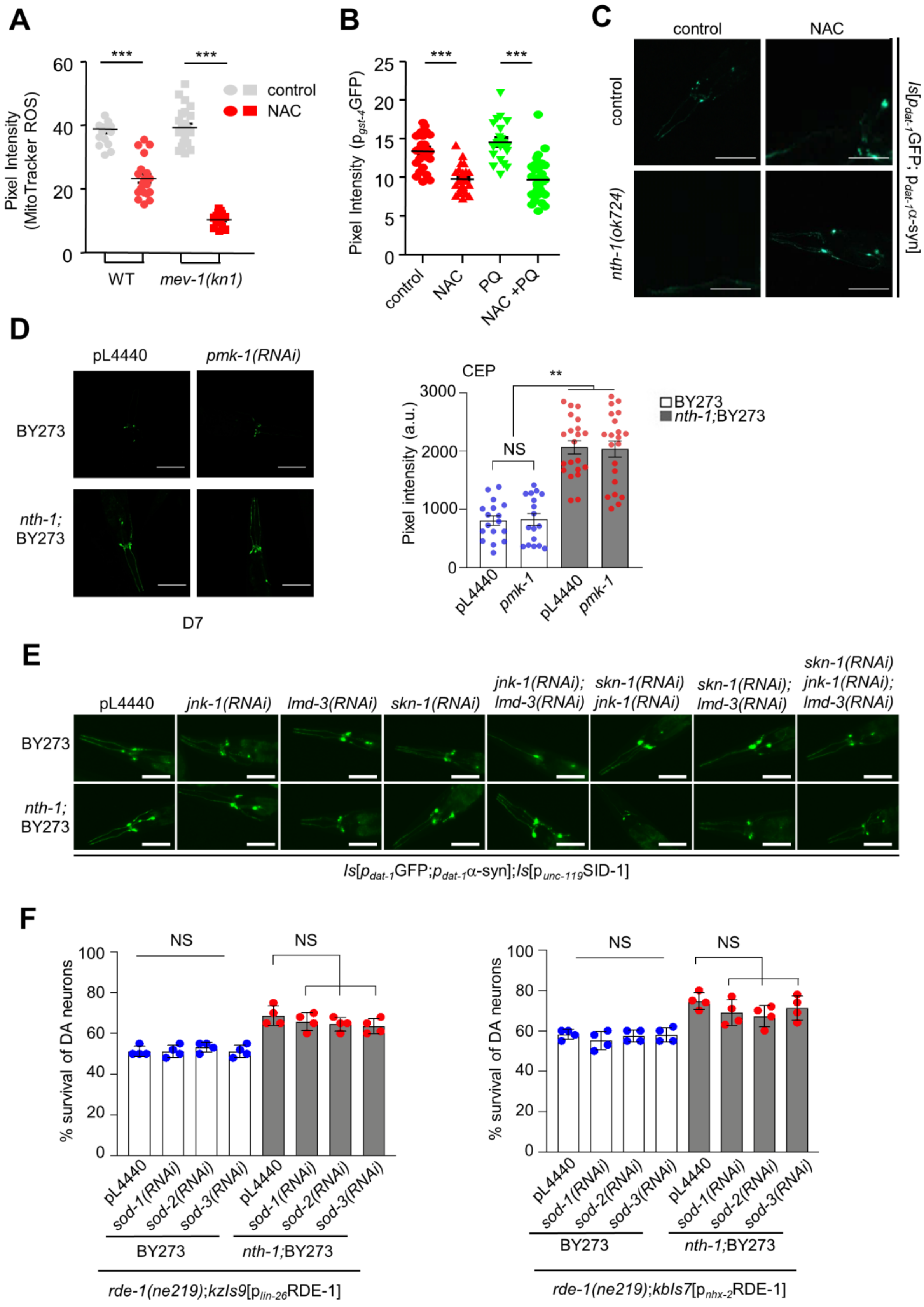

A
Pixel Intensity (MitoTracker ROS)
control
NAC
WT
mev-1(kn1)
B
Pixel Intensity (p_{gst-4}GFP)
control
NAC
PQ
NAC +PQ
C
control
NAC
nth-1(ok724)
Is[p_{dat-1}GFP; p_{dat-1}α-syn]
D
pL4440
pmk-1(RNAi)
BY273
nth-1; BY273
D7
CEP
Pixel intensity (a.u.)
BY273
nth-1;BY273
pL4440
pmk-1
E
pL4440
jnk-1(RNAi)
lmd-3(RNAi)
skn-1(RNAi)
jnk-1(RNAi); lmd-3(RNAi)
skn-1(RNAi) jnk-1(RNAi)
skn-1(RNAi); lmd-3(RNAi)
skn-1(RNAi) jnk-1(RNAi); lmd-3(RNAi)
BY273
nth-1; BY273
Is[p_{dat-1}GFP;p_{dat-1}α-syn];Is[$p_{unc-119}$SID-1]
F
% survival of DA neurons
pL4440
sod-1(RNAi)
sod-2(RNAi)
sod-3(RNAi)
BY273
nth-1;BY273
rde-1(ne219);kzIs9[p_{lin-26}RDE-1]
rde-1(ne219);kbIs7[p_{nhx-2}RDE-1]

Supplementary Figure 5. A mitohormetic response is induced in NTH-1 deficient animals. Related to Figure 3 and 4. (A) NAC supplementation reduces mitochondrial generated ROS in both WT and *mev-1(kn1)* nematodes (n= 30; ****p*< 0.001; unpaired *t*-test). **(B)** SKN-1 activity is diminished in both basal and oxidative stress conditions induced by paraquat (PQ) upon NAC administration (n=40 nematodes per group; ****p*<0.001; unpaired *t*-test). **(C)** BY273 and *nth-1*;BY273 nematodes were treated with NAC. NAC supplementation abolished the neuroprotective effect of NTH-1 deficiency. **(D)** Downregulation of *pmk-1* does not abolish neuroprotection in *nth-1*;BY273 animals (n=15-30 animals; NS *p*>0.05, ***p*<0.01, ****p*<0.001; one-way ANOVA followed by Bonferroni's multiple comparison test). **(E)** Age dependent neuroprotection in *nth-1*;BY273 nematodes is abolished upon depletion of *jnk-1, lmd-3* and *skn-1* and co-depletion of *jnk-1/lmd-3, skn-1/jnk-1 and skn-1/lmd-3* and simultaneous depletion of *skn-1/jnk-1/lmd-3* by RNAi. **(F)** The survival of DA neurons of BY273 and *nth-1;*BY273 nematodes expressed in hypodermis and intestine remains unchanged upon knockdown of *sod-1,sod-2 and sod-3* RNAi (n= 4 biological replicates, 40 to 55 nematodes per group; NS, *p*>0.05 and ****p*<0.001; one-way ANOVA followed Bonferroni's multiple comparison test). Error bars, s.e.m.

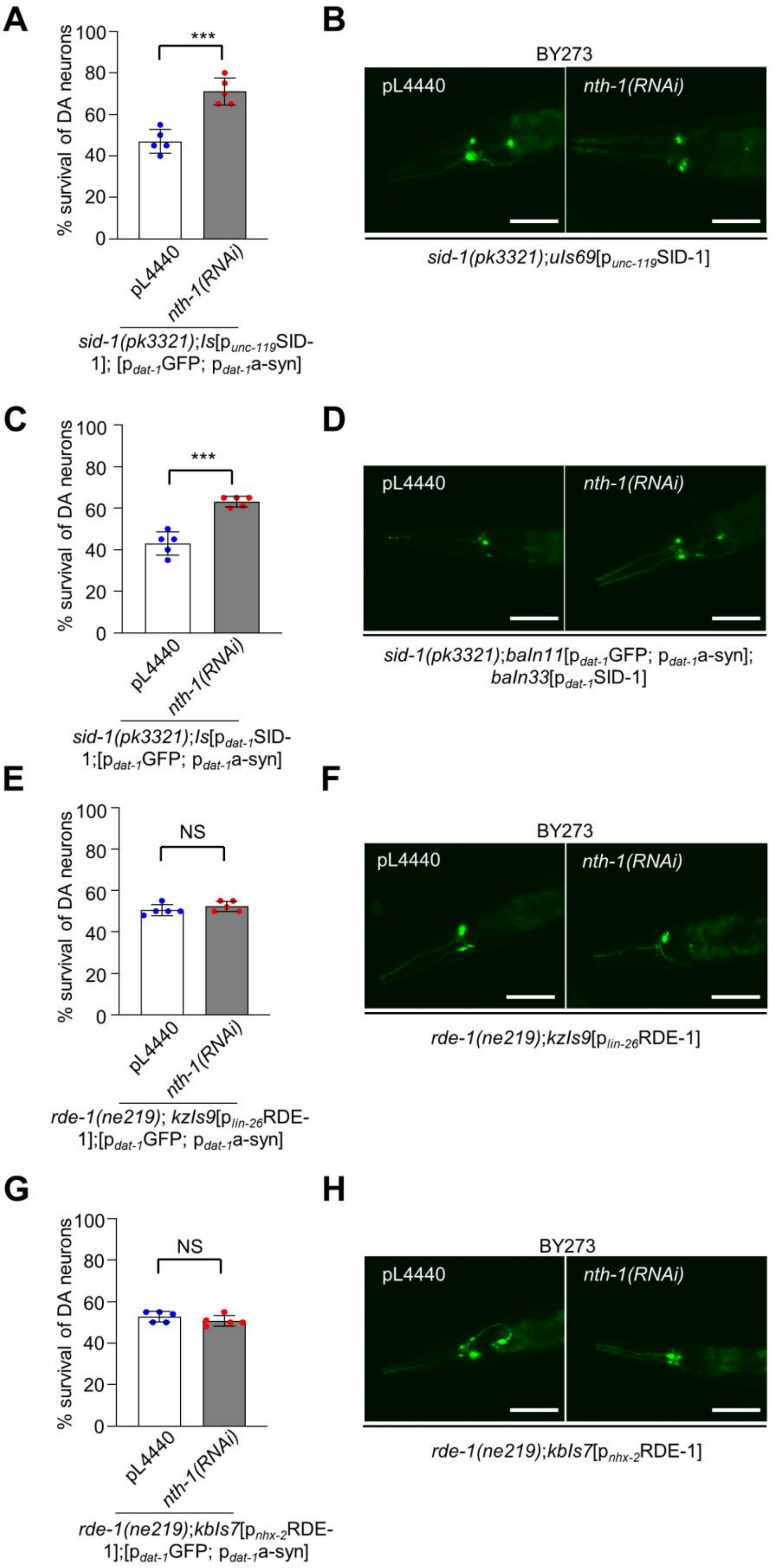

A
B
% survival of DA neurons
100
80
60
40
20
0
***
pL4440
nth-1(RNAi)
sid-1(pk3321);Is[p$_{unc-119}$SID-1]; [p$_{dat-1}$GFP; p$_{dat-1}$a-syn]
BY273
pL4440
nth-1(RNAi)
sid-1(pk3321);uIs69[p$_{unc-119}$SID-1]
C
D
% survival of DA neurons
***
pL4440
nth-1(RNAi)
sid-1(pk3321);Is[p$_{dat-1}$SID-1;[p$_{dat-1}$GFP; p$_{dat-1}$a-syn]
pL4440
nth-1(RNAi)
sid-1(pk3321);baIn11[p$_{dat-1}$GFP; p$_{dat-1}$a-syn];
baIn33[p$_{dat-1}$SID-1]
E
F
% survival of DA neurons
NS
pL4440
nth-1(RNAi)
rde-1(ne219); kzIs9[p$_{lin-26}$RDE-1];[p$_{dat-1}$GFP; p$_{dat-1}$a-syn]
BY273
pL4440
nth-1(RNAi)
rde-1(ne219);kzIs9[p$_{lin-26}$RDE-1]
G
H
% survival of DA neurons
NS
pL4440
nth-1(RNAi)
rde-1(ne219);kbIs7[p$_{nhx-2}$RDE-1];[p$_{dat-1}$GFP; p$_{dat-1}$a-syn]
BY273
pL4440
nth-1(RNAi)
rde-1(ne219);kbIs7[p$_{nhx-2}$RDE-1]

Supplementary Figure 6. Tissue specific and cell autonomous function of NTH-1 to mediate DA neurodegeneration in PD nematode model. Related to Figure 4. Pan-neuronal **(A, B)** and dopaminergic neuron **(C, D)** specific downregulation of NTH-1 protects against α-syn mediated neurodegeneration. Hypodermis **(E, F)** and intestine **(G, H)** specific *nth-1(RNAi)* does not rescue DA neurodegeneration in BY273 nematodes (n= 5 biological replicates, 40 nematodes per group; NS $p>0.05$ and ***$p<0.001$; unpaired t-test). Error bars, s.e.m.

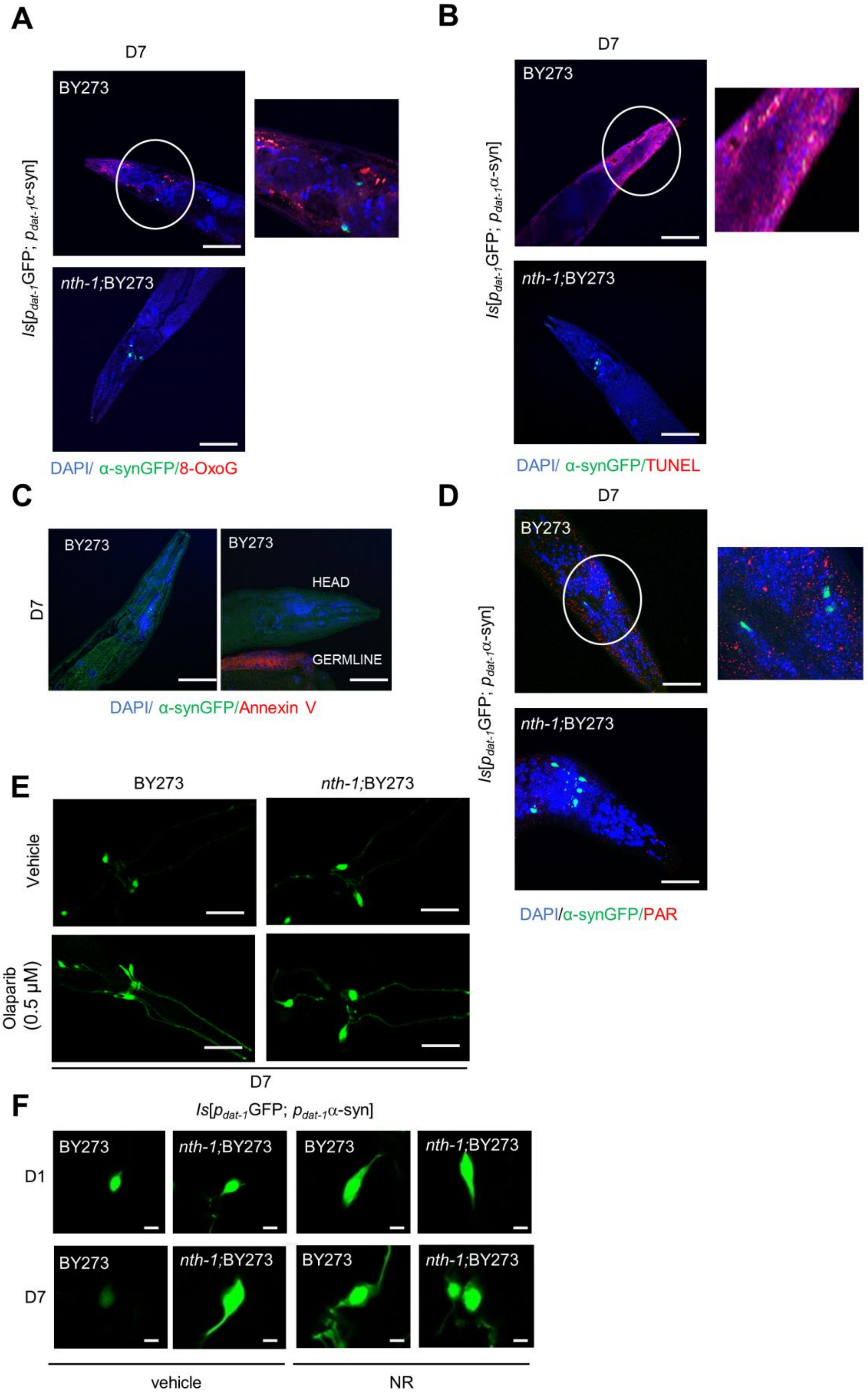

A
D7
BY273
nth-1;BY273
Is[pdat-1GFP; pdat-1α-syn]
DAPI/ α-synGFP/8-OxoG
B
D7
BY273
nth-1;BY273
Is[pdat-1GFP; pdat-1α-syn]
DAPI/ α-synGFP/TUNEL
C
D7
BY273
BY273
HEAD
GERMLINE
DAPI/ α-synGFP/Annexin V
D
D7
BY273
nth-1;BY273
Is[pdat-1GFP; pdat-1α-syn]
DAPI/α-synGFP/PAR
E
BY273
nth-1;BY273
Vehicle
Olaparib (0.5 μM)
D7
F
Is[pdat-1GFP; pdat-1α-syn]
D1
D7
BY273
nth-1;BY273
vehicle
NR

Supplementary Figure 7. DNA damage and BER intermediates accumulate in ageing nematodes. Related to Figure 5. (A) Confocal images showing increase in positive 8-oxo G immunostaining in the head regions of old BY273 animals. Scale bar 20μm; zoomed image, 5μm. **(B)** Confocal images showing increase in TUNEL positive staining in the head regions of old BY273 transgenic animals. Scale bar 20μm; zoomed image, 5μm. **(C)** Positive Annexin V staining present in the germline and absent in the head region of BY273 animal. Scale bar, 20μm. **(D)** Confocal images showing increase in positive PAR staining in the head regions of old BY273 animals. Scale bar 20μm, zoomed image: 5 μm. **(E)** The age dependent neurodegeneration in BY273 is abolished in response to olaparib. It has no further beneficial effect on *nth-1*;BY273 animals. Scale bar, 20μm. **(F)** NR improves DA neuron survival in old BY273 animals. Whereas, NR shows no further benefit in *nth-1*;BY273 animals. Scale bar, 5μm.

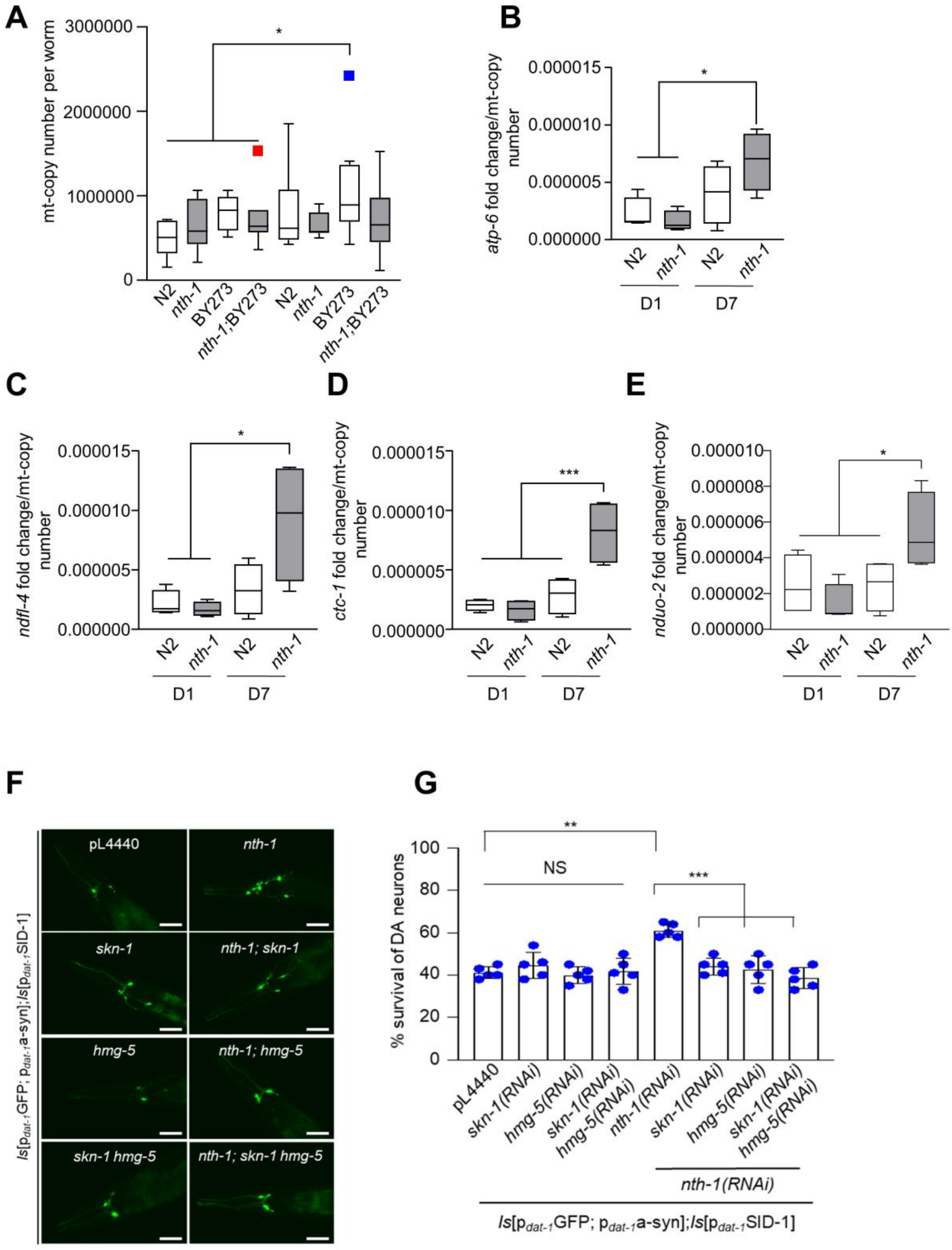
A
mt-copy number per worm
3000000
2000000
1000000
0
N2
nth-1
BY273
nth-1;BY273
B
atp-6 fold change/mt-copy number
0.000015
0.000010
0.000005
0.000000
D1
D7
C
ndfl-4 fold change/mt-copy number
D
ctc-1 fold change/mt-copy number
E
nduo-2 fold change/mt-copy number
F
pL4440
nth-1
skn-1
nth-1; skn-1
hmg-5
nth-1; hmg-5
skn-1 hmg-5
nth-1; skn-1 hmg-5
G
% survival of DA neurons
NS
skn-1(RNAi)
hmg-5(RNAi)
nth-1(RNAi)
Is[pdat-1GFP; pdat-1a-syn];Is[pdat-1SID-1]

Supplementary Figure 8. Mitochondrial mass is unaltered in NTH-1 deficient PD nematodes. Related to Figure 6. (A) The mitochondrial copy number is unaltered in individual animals measured using droplet PCR in young and old N2, *nth-1*, BY273 and BY273;*nth-1* PD animals (n= 8 animals per group, three biological replicates; *p>0.05; one-way ANOVA followed by Tukey's multiple comparison test). **(B-E)** The expression of mitochondrial specific gene *atp-6*, *ndfl-4*, ctc-*1* and *nduo-2* expression normalized to mitochondrial copy number is elevated in old *nth-1* nematodes (n= 8 animals per group, three biological replicates; **p<0.01, ***p<0.001; one-way ANOVA followed by Tukey's multiple comparison test). **(F) (G)** The dopaminergic neuron specific downregulation of *skn-1*, *hmg-5* and co-depletion of *skn-1*,*hmg-5* abrogates neuroprotection in *nth-1* depleted animals in BY273 background. (n= 5 biological replicates; 40 to 55 nematodes per group; NS **p<0.01, ***p< 0.001; one-way ANOVA followed Bonferroni's multiple comparison test). Error bars, s.e.m.

NTH-1 vs. N2	Gene	Fold Change
**Redox**	*acdh-1*	3.28
	gsto-1	3.23
	hphd-1	2.65
	sod-5	2.32
	gst-4	2.31
	acdh-12	2.2
	gst-9	2.08
	gst-6	1.97
	dnj-27	1.88
	sod-3	1.84
	gst-5	-1.8
	sqrd-1	-2.54
	gpx-6	-2.55
	gst-10	-2.69
	gst-3	-2.9

NTH-1 vs. N2	Gene	Fold Change
**SKN-1 interactors**	*ugt-16*	3.13
	C32H11.4	2.72
	cyp-13A6	2.7
	Y102A11A.3	2.44
	gst-4	2.31
	cyc-1	2.23
	Y71H10B.1	2.2
	cyp-13B1	2.2
	cul-4	2.17
	bli-3	2.05
	sur-5	2.03
	C32H11.3	1.93
	cct-2	1.89
	rpn-2	1.89
	sod-3	1.84
	ogt-1	1.82
	lgc-1	1.81
	skr-5	-2.13
	rhy-1	-2.14
	B0024.4	-2.16
	fbxa-182	-2.16
	T26F2.2	-2.2
	Y6E2A.4	-2.45
	sqrd-1	-2.54
	osm-11	-2.55
	gst-10	-2.69
	nekl-2	-2.69
	ins-7	-3
	H20E11.3	-3.7
	hsp-16.2	-6.93

Supplementary Table 1 Comparative results in change of expression of redox and SKN-1 regulated genes, from mixed stages populations of wild type N2 vs *nth-1(ok724)*. Related to Figure 3 and 4.

NTH-1 vs. N2	Gene	Fold Change
**DAF-16 interactors**	C35C5.8	6.75
	hsp-17	4.16
	sodh-1	3.66
	unc-2	3
	unc-10	2.83
	unc-13	2.63
	let-363	2.33
	sod-5	2.32
	gst-4	2.31
	H03A11.2	2.24
	Y71H10B.1	2.2
	nhr-21	2.2
	C17H12.8	2.18
	Y45F10D.6	2.11
	daf-15	2.06
	sgt-1	2.05
	sur-5	2.03
	cct-1	2.01
	drp-1	1.96
	ruvb-1	1.92
	cct-2	1.89
	fkb-5	1.88
	sod-3	1.84
	ogt-1	1.82
	hlh-29	-1.85
	pha-4	-1.86
	unc-43	-1.98
	spp-1	-2.06
	ins-1	-2.17
	tni-3	-2.32
	hsp-16.11	-2.53
	mtl-1	-2.53
	ins-7	-3
	lys-7	-3.4
	ftn-1	-3.49
	ZC204.12	-3.75
	hlh-13	-3.75
	cyp-35B1	-3.83
	ins-35	-4.32
	hsp-16.48	-5.58
	hsp-16.2	-6.93
	cpr-2	-13.9

Supplementary Table 2 Comparative results in change of expression of DAF-16 regulated genes, from mixed stages populations of wild type N2 vs *nth-1(ok724)*. Related to Figure 3 and 4.

Gene	No_variants	Minor_allele_count_PD	Minor_allele_count_control	SKATO_p	SKATO_p_Bonferroni
MUTYH	2	2	1	0,415	1,000
MPG	0	0	0	NA	NA
UNG	0	0	0	NA	NA
OGG1	8	5	10	0,561	1,000
NTHL1	5	5	3	0,493	1,000
NEIL1	1	1	1	0,761	1,000
NEIL2	1	6	0	0,003	0,049
APEX1	0	0	0	NA	NA
POLG	10	18	19	0,048	0,667
FEN1	0	0	0	NA	NA
PNKP	4	5	5	0,623	1,000
EXOG	1	2	2	0,796	1,000
LIG3	2	1	1	0,288	1,000
ERCC6	12	8	12	0,630	1,000
ERCC8	4	5	2	0,204	1,000
YBX1	0	0	0	NA	NA
NUDT1	2	0	2	0,561	1,000
PRIMPOL	2	0	2	0,770	1,000
SSBP1	1	1	0	0,663	1,000

Supplementary Table 3 BER is a susceptibility modifier in PD. Related to Figure 1 and 7.

Genetic variants in selected BER genes in PD patients versus controls as described (Gaare et al., 2018). Genetic associations between individual variants and the PD phenotype were calculated by the SKAT, sequence kernel association test. A significant enrichment of genetic variation in BER genes was found at the pathway level (SKAT-O: p= 0.03). This effect was largely driven by *NEIL2,* a DNA glycosylase that removes oxidized pyrimidines from DNA and has a substrate specificity similar to that of NTH-1. Analyses on the single gene level revealed significant enrichment (p=0.049, after Bonferroni correction) of a single variant, (rs150931138), which introduces a Gly26Ala missense mutation in NEIL2. This variant was detected in 6/411 PD cases and 0/640 controls.
